# From efficiency to sustainability: organic additives for interfacial regulation in lithium metal batteries

**DOI:** 10.1039/d5sc03975d

**Published:** 2025-09-15

**Authors:** Wei Gu, Di He, Yuting Qin, Chongchong Fu, Jiahui Lu, Tianyi Wang, Guoxiu Wang, Bing Sun

**Affiliations:** a School of Chemistry and Chemical Engineering, Yangzhou University Yangzhou 225002 P. R. China Wangty@yzu.edu.cn; b Centre for Clean Energy Technology, School of Mathematical and Physical Sciences, Faculty of Science, University of Technology Sydney Sydney NSW 2007 Australia Guoxiu.wang@uts.edu.au Bing.Sun@uts.edu.au

## Abstract

The utilization of lithium (Li) metal anodes is gaining renewed attention due to the increasing demand for electric vehicles (EVs) and the continuous rise in traditional energy consumption. These Li metal anodes exhibit a high theoretical discharge capacity (3860 mAh g^−1^) and an ultra-low redox potential (−3.04 V *vs.* the SHE), making them the coveted “Holy Grail” for next-generation lithium-ion batteries (LIBs). However, challenges in terms of uncontrolled formation of Li dendrites, instability in the solid electrolyte interphases (SEI) layer, and numerous parasitic reactions have hindered the commercialization of Li metal anodes. In recent years, extensive research has been conducted on the appropriate utilization of organic additives as a long-term, stable, cost-effective, and practical approach to enhancing their stability. The present review investigates the effects of various types and molecular weights of organic additives on the preservation of Li metal anodes and their influence on SEI membrane modification. Finally, we offer valuable insights into the prospective development trajectory of organic additives that are compatible with Li metal anodes.

## Abstract 2

Schematic diagram of the recent progress in regulating solid electrolyte interphases with organic additives to achieve high-performance lithium metal batteries.

## Graphical abstract

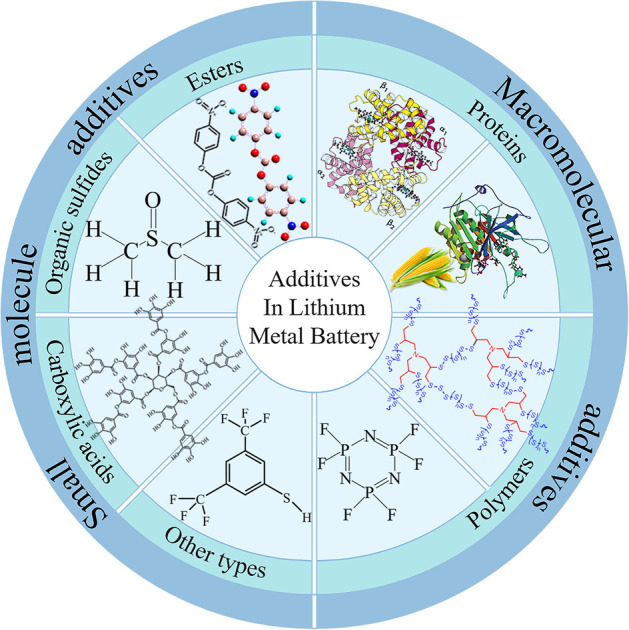

## 1. Introduction

**Fig. 1:**
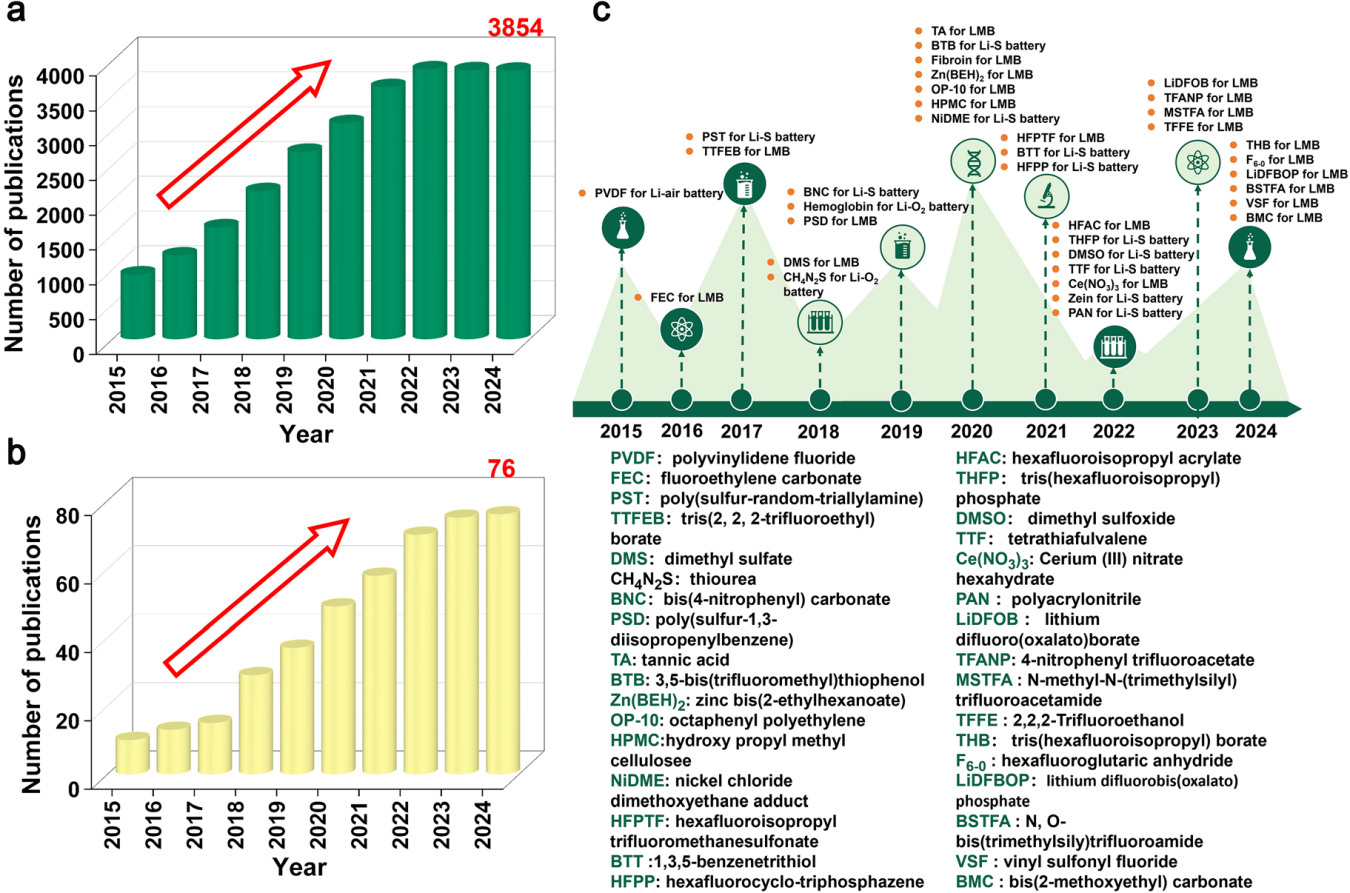
The comparison of scientific publications based on the search keyword (a) “lithium metal anode” and (b) “lithium metal anode” & “electrolyte additives”, as retrieved through the Web of Science database, covering data reported in the past ten years (2015–2024). (c) Relevant breakthroughs have been made in the utilization of organic additives as electrolyte supplements for lithium metal battery research reported in the past ten years (2015–2024).

As the effects of climate degradation and the rapid depletion of fossil fuels continue to worsen,^1,2^ there is a growing need for new energy technologies that can effectively utilize lithium-ion batteries (LIBs) as energy storage carriers.^3,4^ The traditional LIBs, which utilize graphite as the anode electrode, possess inherent limitations that impede their ability to achieve the theoretical capacity and energy density required for electric vehicles (EVs) and large-scale energy storage stations (LESSs).^5,6^ Consequently, there is a growing interest in a more direct and dynamic anode electrode option,^7,8^ namely Li metal anodes (Fig. 1a). Conventional batteries used in rocking chairs employ intercalated anode electrode materials, whereas the lithium metal negative electrode relies on pure metal Li for storage.^9–11^ The latter exhibits an exceptionally high theoretical capacity of 3860 mAh g^−1^ and the lowest relative electrode potential of −3.04 V *vs.* the SHE, making it highly suitable for the development of lightweight and compact LIBs.^12–14^ Furthermore, its low relative electrode potential positions it as a prime candidate for the power battery of the next generation of EVs.^15,16^ Consequently, lithium metal negative electrodes, especially ultra-thin ones, are widely regarded as the optimal choice for the anode electrodes of high energy density LIBs in the future.^17–20^

The utilization of Li metal anodes can be traced back to the inception of the first lithium-ion battery in the 1970s, which was pioneered by Dr M. S. Whittingham and employed Ti_2_S as the cathode alongside Li metal as the anode.^21–23^ The practical implementation of Li metal anodes has been significantly restricted due to numerous inherent flaws.^24–26^ The utilization of graphite anodes instead of Li metal anodes has been prevalent for several decades. The challenges faced in the practical implementation of lithium metal anodes are outlined below.^27^ Foremost, the uncontrollable proliferation of Li dendrites poses a significant challenge for numerous alkali metal anodes during the charging process.^28^ The limited lithium affinity of most anode electrodes in combination with the utilization of copper collectors can cause irregular Li-ion deposition on copper foil.^29,30^ This often leads to the occurrence of Li imperfections, which subsequently lead to an irregular dispersion of the electric field, facilitating the development of Li dendrites. The growth of lithium dendrites is an important reason for battery short circuits, thermal runaway and battery combustion.^31^ Compounding this issue, the exfoliation of “dead Li” emerges when dendrites grow unchecked.^32^ When Li dendrites grow without external intervention like additives, the tips become more active and cause the center of the dendrites to shift upwards. However, after a certain stage of dendritic growth, the connection between the dendrites and the Li metal substrate may break or dissolve due to surface potential energy, resulting in “dead Li” or “exfoliated Li”. The irreversible formation of dead Li leads to a significant loss of active materials and capacity decline, posing a particular challenge for the future development of low-load anodes such as ultra-thin Li metal anodes. Further complicating matters, the unstable solid electrolyte interphase (SEI) layer plays a bad role.^33^ The SEI layer is primarily formed through the reduction and decomposition of electrolytes at the Li metal interface. Depending on the type of electrolyte used, the SEI layers can have different chemical compositions, leading to unique properties. However, the traditional SEI layer is rigid, making it prone to rupture during Li metal expansion. As a result, the SEI layer becomes thicker, the internal resistance of the battery increases, and dendrite growth becomes more likely due to repeated rupture and healing. Finally, parasitic reactions present a multifaceted threat.^34,35^ The Li metal anode displays a significant level of electrochemical reactivity, leading to the decomposition of the electrolyte and gas production within the battery. This can lead to potentially dangerous safety hazards. Moreover, dendrites can significantly and reversibly increase the specific surface area of Li metal anodes, thereby exacerbating side reactions and resulting in a decrease in battery capacity.

**Fig. 2:**
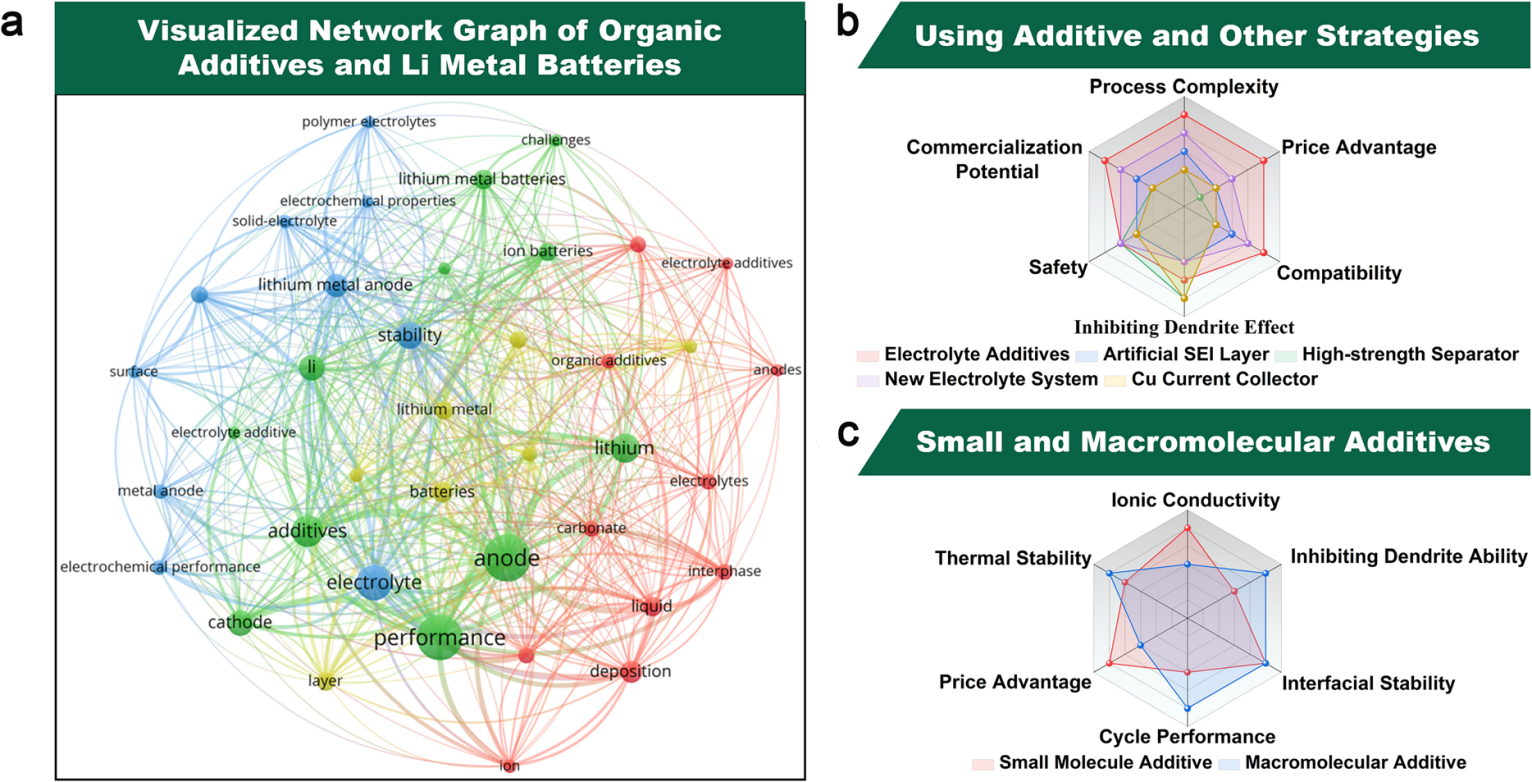
(a) A network visualization graph of high-frequency keywords “additives”, “electrolytes”, and “lithium metal anodes” during the recent 5 years (2020–2024) (data collected from Web of Science and analyzed by VOSviewer). (b) Comparison of interfacial protection strategies for Li metal anodes. (c) Advantages of organic additives.

Strategies for suppressing Li dendrites and protecting interfaces in Li metal anodes can be divided into the following categories: (i) design of new Cu collectors;^36,37^ (ii) adding electrolyte additives;^38–40^ (iii) interface pretreatment (artificial SEI layer);^41,42^ (iv) design of high-strength separators;^43,44^ (v) design of new electrolyte systems.^45,46^ Among these, the utilization of electrolyte additives stands out as the most straightforward, highly efficient, and cost-effective approach (Fig. 1b and 2b).^47,48^ According to the statistical data from the literature published in the past 5 years (searched through Web of Science) and analyzed using VOSviewer, a visualization network displays the high-frequency keywords appearing in the literature and their associations (Fig. 2a). It is evident that there is a close association between additives, electrolytes, and lithium metal anodes. In 2013, Zhang *et al.* pioneered the utilization of low concentrations of selected cations (cesium and rubidium ions) as additives to achieve long-term protection and self-healing of Li metal anodes.^49,50^ With the development of traditional inorganic additives, the use of organic additives has also shown great development prospects (Fig. 1c). The advantages of organic additives are as follows (Fig. 2c). The primary advantage lies in their higher solubility. Due to their polar structures, organic additives exhibit superior solubility and compatibility in Li metal anode electrolytes, such as carbonate-based and ether-based systems. Building upon this, their higher adaptability arises from the structural similarity between organic additives and the main components of the SEI layer (*e.g.*, Li alkyl carbonates (ROCOOLi) and polyethers (ether oligomers)), enabling stronger bonding with the SEI layer, which is critical for enhancing its mechanical and electrochemical properties. Furthermore, compared to structurally simple inorganic additives, organic additives demonstrate higher designability through purposeful functional group modifications,^51,52^ offering unique advantages in designing complex electrolyte systems (*e.g.*, high-voltage, flame-retardant, and low-temperature electrolytes). Additionally, organic additives are more environmentally benign, as they avoid heavy metal ions (*e.g.*, Cr^3+^, Ba^2+^, Cs^+^, Rb^+^ and In^3+^) commonly used in inorganic counterparts, thereby reducing environmental and health risks during battery production and recycling. Lastly, their cost-effectiveness stands out: organic additives achieve equivalent SEI regulatory effects at lower doses compared to bulk inorganic additives, significantly reducing overall preparation costs. Due to these advantages, organic additives for Li metal anodes currently account for 60% of electrolyte additive research and development.

**Fig. 3:**
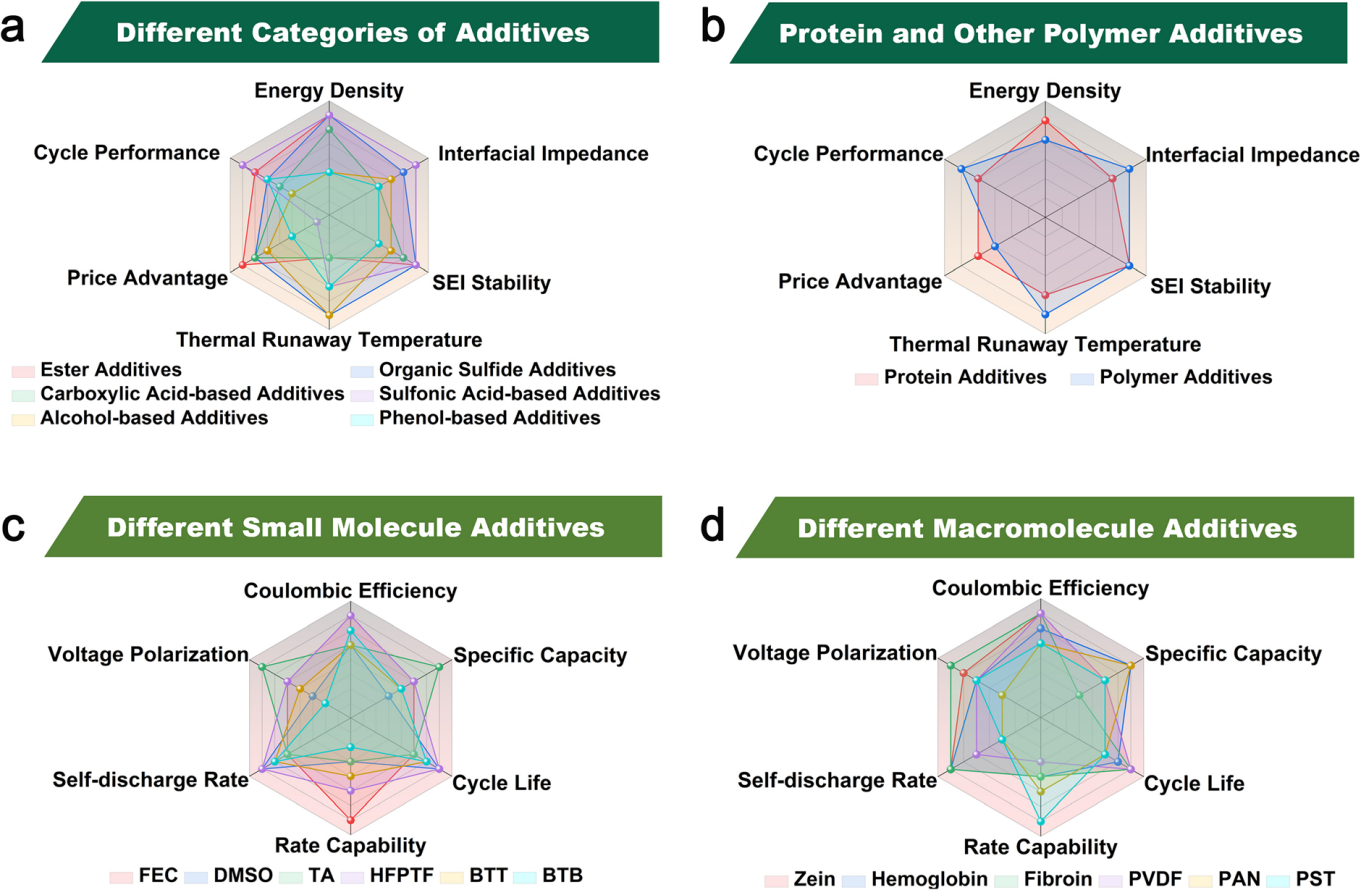
(a) Macroscopic comparison of properties of small molecule additives. (b) Macroscopic comparison diagram of the properties of macromolecular additives. (c) Micrographic comparison of properties of small molecule additives. (d) Micrographic comparison of properties of macromolecular additives.

Considering the critical role solvent systems play in determining the effectiveness of electrolyte additives, this review first provides a macro-level discussion based on different solvent environments, including carbonate-based electrolytes, ether-based electrolytes, and other specialized solvent systems. Subsequently, from a micro-level perspective, we systematically introduce recent advances in organic small-molecule and macromolecule additives categorized by their molecular types, closely examining the interactions between these additives and their respective solvent systems to enhance lithium metal battery (LMBs) performance. Specifically, for organic small-molecule additives, we emphasize their applications in lipid compounds, organic sulfides, carboxylic acid compounds, and other relevant small molecules (Fig. 3a). Regarding organic macromolecule additives, we mainly discuss how their degree of polymerization affects interfacial reactions under high current-density plating conditions (Fig. 3b). Additionally, aspects such as cost-effectiveness, practical applicability, and environmental sustainability are comprehensively considered throughout this review (Fig. 3c and d).

## 2. Different solvent systems

The choice of solvent systems critically influences the physicochemical properties, interfacial chemistry, and electrochemical performance of electrolyte additives in LMBs. Organic additives often exhibit significantly varied interactions and functional mechanisms within different solvents, which directly impact the stability and composition of the SEI, the uniformity of lithium deposition, and ultimately, battery safety and cycling performance. In this section, we systematically analyze four major categories of solvent systems commonly used in lithium metal batteries: carbonate-based electrolytes, ether-based electrolytes, solid-state or GPEs, and specialized solvent systems. We specifically highlight how each solvent environment affects additive solubility, additive decomposition pathways, SEI layer formation, and their subsequent implications for the suppression of lithium dendrites and parasitic reactions. This comprehensive understanding provides essential guidance for the rational selection and optimization of electrolyte additives tailored to specific solvent systems, thus advancing the practical application and commercialization of lithium metal batteries.

### 2.1. Carbonate-based electrolytes

Carbonate-based solvents are among the most widely utilized electrolyte solvents in commercial lithium-ion and lithium metal batteries due to their high dielectric constants, excellent lithium salt solubility, and favorable electrochemical stability.^53^ Typical solvents in this category include ethylene carbonate (EC), dimethyl carbonate (DMC), diethyl carbonate (DEC), ethyl methyl carbonate (EMC), and propylene carbonate (PC).^54,55^ These solvents exhibit distinct physicochemical properties that make them particularly suitable for lithium-ion batteries. Specifically, EC possesses a high dielectric constant (∼90 at 25 °C) that effectively dissociates lithium salts, leading to high ionic conductivity. Linear carbonates such as DMC, DEC, and EMC, characterized by lower viscosity and melting points, are typically employed in combination with cyclic carbonates like EC to enhance ionic mobility and overall electrolyte performance.^56^

However, despite these advantages, carbonate-based electrolytes present several intrinsic limitations, particularly when used with lithium metal anodes. Primarily, carbonate solvents exhibit relatively high reactivity with lithium metal, which can lead to continuous electrolyte decomposition and unstable SEI formation.^57,58^ This often results in rapid growth of lithium dendrites and reduced coulombic efficiency, significantly impairing the battery's cycle life and safety. Moreover, carbonate solvents, particularly EC and PC, have high melting points and viscosities, which can negatively affect electrolyte ionic conductivity at low temperatures, restricting their application range under extreme conditions.^59,60^

To mitigate these inherent drawbacks, the introduction of organic electrolyte additives has been extensively studied. Organic additives, such as ester derivatives (*e.g.*, fluoroethylene carbonate (FEC) and vinylene carbonate (VC)), have shown high solubility and excellent compatibility within carbonate-based solvents. Due to structural similarity and polarity alignment between additives and solvents, these additives are able to effectively modify the SEI composition and morphology, enhancing SEI stability and lithium-ion transport capability. Specifically, additives like FEC significantly facilitate the formation of lithium fluoride (LiF)-enriched SEI layers, offering improved mechanical integrity and chemical stability, thereby reducing dendrite formation and electrolyte decomposition. Wang *et al.* developed a gel-polymer electrolyte using pure FEC as the solvent and showed that FEC substantially improves interfacial chemistry for Li metal at high voltages in 2025.^61^ In their design, a co-monomer, pentafluorophenyl methacrylate (PFPMA), was included to prevent FEC's downside, namely, the generation of HF at high voltage, by forming a mixed SEI with lithium difluoro(oxalato)borate (LiDFOB) that suppresses FEC decomposition. The result was a highly conductive, LiF-rich SEI and a Li-ion transference number of 0.87, enabling stable cycling of an NMC811 cathode at 4.5 V with a Li metal anode. These findings underscore FEC's unique role: it positively impacts interfacial chemistry and supports fast Li-ion transport, although complementary strategies (like co-additives or polymers) can mitigate its side reactions under extreme conditions. In 2025, Wagner-Henke *et al.* specifically investigated VC in a Li–metal system, finding that VC-modified electrolytes form a denser, more uniform SEI on Li metal anodes.^62^ This reinforced SEI leads to smoother Li deposition morphology and better cycling stability compared to VC-free electrolytes, highlighting VC's role in enhancing interfacial stability on lithium metal. Indeed, the VC additive helps “understand the SEI formation, morphology and stability” on Li anodes, corroborating its protective effect in carbonate systems. The interaction of VC with carbonate solvents also modulates the solvation structure of lithium ions, increasing the fraction of lithium salt anions within the solvated complexes, and thus elevating lithium-ion transference numbers and ionic conductivity.

Despite their widespread usage and significant improvements facilitated by additives, carbonate-based electrolyte systems still face safety and environmental challenges. Their relatively high flammability and volatility raise safety concerns under thermal abuse conditions. Additionally, traditional carbonate solvents and additives often pose environmental and recycling challenges due to their chemical stability and potential toxicity. Consequently, research efforts are actively directed toward the development of environmentally friendly and inherently safe carbonate-based solvents and additives. Bio-derived carbonate solvents and novel carbonate derivatives with lower flammability and better environmental degradability have recently emerged as promising directions to address these sustainability issues. Overall, understanding the unique interactions between carbonate-based solvents and various organic additives is critical for optimizing electrolyte formulations that enhance lithium metal battery performance. Future advancements in additive chemistry and solvent engineering are expected to further overcome the current limitations, promoting broader and safer utilization of carbonate-based electrolyte systems in next-generation lithium metal batteries.

### 2.2. Ether-based electrolytes

Ether-based solvents have garnered significant attention in recent years as promising alternatives to conventional carbonate solvents, particularly in the context of lithium metal batteries.^63^ These solvents are characterized by their low viscosity, high lithium metal compatibility, and relatively low reduction potentials, making them especially suitable for stabilizing reactive lithium surfaces. Typical ether-based solvents include 1,2-dimethoxyethane (DME), 1,3-dioxolane (DOL), tetrahydrofuran (THF), and glymes such as diethylene glycol dimethyl ether (DEGDME) and tetraethylene glycol dimethyl ether (TEGDME).^64,65^ These solvents are often used individually or in binary mixtures to tailor electrolyte properties such as solvation structure, ionic conductivity, and electrochemical stability.^66^ In terms of physicochemical characteristics, ether solvents generally exhibit moderate dielectric constants (∼7–10), sufficient to dissolve a wide range of lithium salts including LiTFSI, LiFSI, and LiNO_3_. Although their dielectric constants are lower than those of carbonate solvents such as EC, ether solvents compensate through strong donor numbers and Lewis basicity, facilitating efficient lithium salt dissociation and stable solvation structures. Additionally, their low viscosities and low melting points enable high ionic mobility and favorable low-temperature performance, which are especially important for fast-charging and cold-climate applications.^67,68^

A key advantage of ether-based solvents lies in their excellent interfacial compatibility with lithium metal. Unlike carbonates, which undergo aggressive reduction upon coming in contact with lithium, ethers typically form passivating interphases that are more chemically stable and mechanically robust. For example, DOL-containing systems can undergo ring-opening polymerization to form polymeric SEI components, contributing to a flexible and uniform lithium-ion diffusion layer. This mitigates dendritic lithium growth and enhances coulombic efficiency during cycling.^69^ Moreover, ethers generally exhibit low reactivity with plated lithium, allowing for smoother lithium deposition and improved cycling life in lithium metal batteries.^70,71^

The compatibility of ether solvents with functional electrolyte additives is another critical factor. Ethers can dissolve a variety of organic and inorganic additives, including LiNO_3_, LiI, and fluorinated ethers or sulfones. The solubility of these additives in ether solvents is typically sufficient to enable effective participation in interfacial chemistry. However, the lower polarity and dielectric constant of ethers compared to carbonates can sometimes limit the dissolution of certain high-polarity additives, potentially necessitating co-solvent systems or cosolubilizing agents. Furthermore, the solvation structures in ether-based electrolytes are often dominated by solvent–Li^+^ interactions rather than anion coordination, which affects both lithium-ion transport properties and the reductive decomposition behavior of additives.^72,73^ From an electrochemical perspective, ether-based electrolytes generally have narrower electrochemical stability windows (∼1.0 to 4.0 V *vs.* Li^+^/Li) than carbonate systems, due to the lower oxidative stability of ether solvents. As a result, they are less suitable for pairing with high-voltage cathodes (*e.g.*, NCM811 or LiNi_0.5_Mn_1.5_O_4_) without further modification or protection strategies. Nonetheless, in applications focused on lithium metal or lithium–sulfur (Li–S) batteries, where lower voltage cutoffs are acceptable, ether electrolytes offer compelling advantages in terms of lithium reversibility and SEI stability.^74^ In terms of safety and environmental sustainability, ether solvents generally exhibit lower flash points and higher volatility compared to carbonates, raising concerns regarding flammability and vapor pressure under thermal abuse conditions. However, their relatively simpler molecular structures and lower chemical persistence offer potential advantages in terms of environmental degradation and recyclability. Research into fluorinated or partially halogenated ethers aims to reduce volatility and improve flame resistance, although often at the cost of increased synthetic complexity and environmental burden.^75^

Despite these merits, ether-based electrolytes also face intrinsic limitations. Their limited oxidative stability constrains the use of high-voltage cathodes unless additional protective strategies, such as cathode surface coatings or high-voltage additives, are implemented.^76^ Moreover, some ether solvents (*e.g.*, DOL) are prone to chemical or electrochemical polymerization, which can lead to increased viscosity or gelation during long-term cycling. Another challenge lies in tuning the solvation structure to balance ionic conductivity, interfacial stability, and transference number, especially under high-concentration or localized-high-concentration electrolyte (LHCE) conditions.^77^

In summary, ether-based electrolytes offer a unique set of properties that make them highly attractive for stabilizing lithium metal anodes and enabling high-performance lithium metal and Li–S batteries. Their favorable lithium compatibility, high ionic mobility, and functional additive adaptability distinguish them from carbonate-based systems. However, addressing their oxidative instability, flammability, and limited voltage range remains critical for their broader application in advanced lithium battery technologies. A comprehensive understanding of the interaction mechanisms between ether solvents and organic additives—particularly how they govern solvation structures and interfacial processes—will be essential for the rational design of next-generation ether-based electrolytes.

### 2.3. Solid-state and gel polymer electrolytes

Solid-state electrolytes (SSEs) and gel polymer electrolytes (GPEs) have garnered significant attention as promising alternatives to conventional liquid electrolytes in lithium metal batteries due to their enhanced safety profiles, mechanical robustness, and potential for improved electrochemical stability.^78,79^ Typical SSEs primarily include inorganic ceramic electrolytes (such as lithium garnets Li_7_La_3_Zr_2_O_12_ and sulfides Li_10_GeP_2_S_12_), solid polymer electrolytes (SPEs, notably polyethylene oxide (PEO)-based), and composite solid electrolytes (CSEs), which combine the advantages of both polymer and inorganic electrolytes.^80,81^ GPEs, on the other hand, typically incorporate polymer matrices such as poly(vinylidene fluoride-*co*-hexafluoropropylene) (PVDF-HFP), poly(methyl methacrylate) (PMMA), or cellulose derivatives swollen with liquid electrolyte solutions. From a physicochemical perspective, SSEs exhibit negligible volatility, low flammability, and exceptional thermal stability. Polymers employed in SPEs and GPEs possess high dielectric constants, which facilitate lithium salt dissociation, ensuring appreciable ionic conductivity.^82,83^ The polymeric matrix provides mechanical flexibility and robustness, crucial for suppressing dendritic lithium growth by mechanically reinforcing the SEI. GPEs further integrate advantageous properties of liquid electrolytes, such as high ionic conductivity and favorable lithium salt solubility, with the mechanical stability and leakage resistance characteristic of solid systems.

Despite these strengths, SSEs and GPEs also exhibit inherent limitations. Ionic conductivity, particularly at room temperature, remains substantially lower compared to liquid counterparts, especially for SPEs like PEO due to their semi-crystalline nature. The interaction between lithium metal anodes and SSEs or GPEs frequently presents interfacial compatibility issues, leading to elevated interfacial resistance, uneven lithium deposition, and increased polarization.^84^ Additionally, solid electrolytes, particularly inorganic ceramics, suffer from brittleness, complicating large-scale manufacturing and integration with lithium metal electrodes. To address these challenges, the introduction of organic additives into SSEs and GPEs has been extensively explored. Organic additives with appropriate solubility and compatibility, such as plasticizers, lithium salts with lower lattice energy, and functionalized small molecules or polymers, can significantly enhance ionic conductivity by reducing polymer crystallinity and promoting lithium salt dissociation.^85^ Moreover, additives that form robust SEI layers enriched with stable inorganic species (*e.g.*, lithium fluoride and lithium sulfide) effectively mitigate interfacial impedance and dendritic growth, substantially improving cycle stability and coulombic efficiency.^86^

Regarding safety and environmental aspects, SSEs and GPEs inherently exhibit superior safety profiles over conventional liquid electrolytes due to reduced flammability and leakage risks. However, the environmental impact of polymer synthesis, potential toxicity of additives, and recyclability of composite materials continue to be pertinent issues. Therefore, recent research is actively investigating environmentally benign and sustainable polymer hosts and additives, such as bio-derived cellulose polymers and biodegradable plasticizers.^87^

In summary, a comprehensive understanding of the interplay between polymeric matrices, lithium salts, and organic additives within SSEs and GPEs is essential to overcoming their intrinsic limitations. Continuous advancements in additive chemistry, electrolyte formulation, and interface engineering are anticipated to substantially enhance the practical applicability of these electrolytes in next-generation lithium metal batteries.

### 2.4. Specialized solvent systems

Specialized solvent systems have emerged as important alternatives to traditional carbonate and ether-based electrolytes, particularly in high-performance LMBs applications. These solvent systems encompass unique categories such as ionic liquids (ILs), fluorinated solvents, nitriles, sulfones, and bio-derived solvents, each exhibiting distinctive physicochemical properties tailored for specific battery performance enhancements.^88^

ILs, such as 1-ethyl-3-methylimidazolium bis(trifluoromethylsulfonyl)imide (EMImTFSI) and *N*-methyl-*N*-propylpyrrolidinium bis(fluorosulfonyl)imide (Pyr13FSI), are notable for their exceptionally high dielectric constants, superior lithium salt solubility, negligible vapor pressure, and broad electrochemical stability windows.^89,90^ These solvents are characterized by their thermal and electrochemical robustness, significantly reducing volatility and flammability concerns typical of traditional organic solvents. However, their relatively high viscosity and density may limit ionic conductivity and practical applicability at lower temperatures. The addition of specific organic additives into IL-based electrolytes can modulate viscosity, enhance lithium-ion transport, and stabilize the lithium metal interface by forming uniform, stable SEI layers.

Fluorinated solvents, such as FEC and fluorinated ethers, provide enhanced chemical stability, wide electrochemical windows, and effective SEI formation capabilities.^90^ Their high dielectric constants enable excellent lithium salt dissociation, promoting high ionic conductivity. Additives in fluorinated solvent systems generally have superior solubility, and their interactions often result in fluoride-rich SEI layers with improved mechanical and chemical stability, effectively inhibiting lithium dendrite growth. Nevertheless, these solvents might introduce environmental concerns due to their persistent and potentially toxic degradation products.^91^

Nitrile-based solvents, such as acetonitrile (ACN), demonstrate high dielectric constants and excellent lithium salt solubility, contributing to increased ionic conductivity and lithium transference numbers.^92,93^ However, their strong reactivity with lithium metal necessitates careful formulation with additives to stabilize the lithium–electrolyte interface and control SEI formation. Organic additives effectively mitigate these drawbacks by reducing electrolyte decomposition and enhancing interface compatibility.

Sulfone solvents, including sulfolane and dimethyl sulfone (DMSO), feature high thermal stability, high dielectric constants, and excellent salt solubility, making them suitable for high-voltage battery applications.^94,95^ Despite these advantages, sulfones typically exhibit high viscosity, restricting ionic conductivity and limiting their low-temperature performance. Incorporating organic additives or co-solvents can effectively address these limitations, improving viscosity and enhancing interface stability with lithium metal.

Bio-derived solvents represent a growing area of interest in developing environmentally friendly and sustainable battery technologies. Examples include ethyl lactate and dimethyl isosorbide, characterized by moderate dielectric constants, good solubility of lithium salts, and favorable biodegradability profiles.^96^ These solvents often demonstrate lower toxicity and improved environmental compatibility compared to traditional solvents. However, their lower electrochemical stability and moderate reactivity with lithium metal may limit their application unless carefully combined with specialized additives to optimize SEI characteristics and battery performance.^97^

Overall, specialized solvent systems combined with targeted organic additives offer promising routes to overcoming intrinsic limitations related to reactivity, solubility, ionic conductivity, and environmental safety, paving the way toward high-performance, safer, and more sustainable lithium metal battery technologies.

## 3. Small molecule additives

Organic small molecules with a molecular weight below 1000 are known as low molecular weight organic additives.^98^ These additives have higher solubility than their larger counterparts and have less of a negative impact on Li-ion conductivity (Table 1).^99^ The lower molecular weight of these additives enhances the molecular dynamics of Li-ion deposition on Li metal anodes, thereby facilitating uniform deposition of Li-ion and reducing the nucleation potential of Li-ion (altering the electric field distribution of Li buds).^100^ Our research delved into the application of small molecule additives in LMBs, with a particular emphasis on safeguarding lithium metal anodes and enhancing overall battery performance. Within this study, we concentrated on four specific directions, namely lipid compounds, organic sulfides, carboxylic acid compounds, and other relevant categories.

**Table 1:** The application of small molecule electrolyte additives in rechargeable batteries

Organic additives	Applications	Cathodes	Anodes	Electrode	Electrolyte	Electrochemical performances	Ref.
HFAC	Lithium metal battery	NCM622	Li	80 wt% NCM622 powder + 10 wt% acetylene black + 10 wt% PVDF	1 M LiPF_6_ in EC/DMC + 1 wt% HFAC	111 mAh g^−1^ at 200 mA g^−1^ after 200 cycles	101
TTFEB	Lithium metal battery	LiFePO_4_	Li	80 wt% LiFePO_4_ + 10 wt% Super-P + 10 wt% PVDF in *N*-methy-l-2-pyrrolidone (NMP)	1 M LiPF_6_ in EC/DM (1 : 2) + 2 wt% TTFEB	133.6 mAh g^−1^ at 170 mA g^−1^ after 500 cycles	102
THFP	Lithium metal battery	NCM622	Li	80 wt% active materials + 10 wt% carbon black + 10 wt% PVDF	1 M LiPF_6_ in PC/EMC/EP (42.5 : 42.5 : 15) + 2 wt% THFP	145 mAh g^−1^ at 100 mA g^−1^ after 200 cycles	103
DMS	Lithium metal battery	NCM	Li	LiNi_1/3_Co_1/3_Mn_1/3_O_2_ cathode sheets	1 M LiPF_6_ in EC/DMC (1 : 1) + 1 wt% DMS	120 mAh g^−1^ after 100 cycles	104
FEC	Lithium metal battery	NMC	Li	80 wt% active material + 10 wt% Super P + 10 wt% PVDF	1 M LiPF_6_ in EC/DEC (1 : 1) + 5 wt% DMS	100.1 mAh g^−1^ at 180 mA g^−1^ after 100 cycles	105
BNC	Lithium metal battery	C/S	Li	80 wt% C/S (76.3% S) + 10 wt% carbon black + 10 wt% PVDF	1 M LiPF_6_ + 0.46 MLiNO_3_ in DME/DOL (1 : 1) + 0.11 BNC	778.7 mAh g^−1^ at 1C after 300 cycles	106
BMC	Lithium metal battery	NCM811	Li	96 wt% NCM811 powder + 2 wt% acetylene black + 2 wt% PVDF in NMP	1.2 M BMC in LiFSI + 0.75 wt% LiNO_3_ + 1 wt% LiDFBOP	156.4 mAh g^−1^ at 0.2C/0.3C after 150 cycles	107
DMIC	Lithium metal battery	NCM622	Li	80 wt% NCM622 powder + 10 wt% Super P + 10 wt% PVDF in NMP	1 M LiPF_6_ in EC/DMC (1 : 1) + 1 wt% DMIC	134.1 mAh g^−1^ at 2C after 500 cycles	108
DMSO	Lithium metal battery	LiFePO_4_	Li	80 wt% LiFePO_4_ + 10 wt% Super P + 10 wt% PVDF in NMP	1 M LiTFSI in DME/DOL (1 : 1) + 5 vol% DMSO	51 mAh g^−1^ at 0.2C at 40 °C	109
CH_4_N_2_S	Lithium–O_2_ battery	MnCo_2_O_4_@Ni	Li	0.52 g MnSO_4_·H_2_O + 0.25 g (CH_3_COO)_2_Co·4H_2_O in a 60 ml of ethyl alcohol/deionized (DI) water (4 : 1) + 0.72 g urea	1 M LiTFSI in TEGDME + 1 M CH_4_N_2_S	250 mAh g^−1^ at 100 mA g^−1^ after 160 cycles	110
TTF	Lithium metal battery	CMK-3/S	Li	80 wt% CMK-3/S (3 : 7) + 10 wt% Super P and 10 wt% PVDF in NMP	0.1 M LiNO_3_ in 1 M LiTFSI/DOL DME (1 : 1) + 0.025 M TTF	509 mAh g^−1^ at 0.5C after 500 cycles	111
LiDFOB	Lithium metal battery	Cu	Li		1 M LiFSI in DME/HFE-347 (1/2 vol%) + 0.02 M LiDFOB	CE of 99.4% at 3 mAh g^−1^ at 0.5 mA g^−1^ after 200 cycles	112
Ce(NO_3_)_3_	Lithium metal battery	LiFePO_4_	Li	80 wt% LiFePO_4_ + 10 wt% Super P + 10 wt% PVDF in NMP	PEO/LiFSI in CH_3_CN + 1% Ce(NO_3_)_3_	133.7 mAh g^−1^ at 0.2C after 300 cycles	113
TA	Lithium metal battery	Li	Li		1 mol L^−1^ LiPF_6_ in EC/DMC/EMC (1 : 1 : 1)	270 h at 1 mAh cm^−2^ at 1 mA cm^−2^	114
HFPTF	Lithium metal battery	NCM811	Li	80 wt% NMC811 + 10 wt% PVDF + 10 wt% acetylene black in NMP	1 M LiPF_6_ in EC/DM (1 : 1) + 1 wt% HFPTF	129 mAh g^−1^ at 100 mA g^−1^ after 150 cycles	115
BTT	Lithium metal battery	S	Li	S in CS_2_	1 M LiTFSI + 0.15 M LiNO_3_ in DOL/DME (1 : 1) + BTT	907 mAh g^−1^ at 1C after 300 cycles	116
BTB	Lithium metal battery	S	Li	30 wt% multi-walled carbon nanotubes + 70 wt% S in NMP	1 M LiTFSI + 2% LiNO_3_ in DOL/DME (1 : 1) + 80 mM BTB	700 mAh g^−1^ at 0.1C after 82 cycles	117
HTCN	Lithium metal battery	NCM811	Li	80 wt% NCM811 powder + 10 wt% acetylene black + 10 wt% PVDF in NMP	1 M LiPF_6_ in EC/DMC (3 : 7) + 0.5 wt% HTCN	175 mAh g^−1^ at 1C after 120 cycles	118
PQA-NO_3_	Lithium metal battery	NCM811	Li	90 wt% NCM811 powder + 5 wt% Super P + 5 wt% PVDF in NMP	DDE + 0.1 M PN	110 mAh g^−1^ at 0.1C after 100 cycles at −60 °C	119
1,3-Dithiane	Lithium metal battery	LiFePO_4_	Li	80 wt% LiFePO_4_ + 10 wt% acetylene black + 10 wt% PVDF in NMP	1 M LiPF_6_ in EC/DMC/EMC (1 : 1 : 1) + 2 wt% 1,3-dithiane	125.4 mAh g^−1^ at 1C after 3300 cycles	120

### 3.1. Ester additives

Ester additives are predominantly employed in carbonate-based electrolytes due to their structural compatibility and effective interaction with polar carbonate solvents. The polar ester groups within these additives facilitate enhanced lithium salt dissociation, resulting in improved ionic conductivity and stabilized SEI layers. The unique capability of ester additives to form robust, uniform, and LiF-enriched SEI layers significantly mitigates lithium dendrite growth and electrolyte decomposition, making them especially suitable for lithium metal battery applications.

**Fig. 4:**
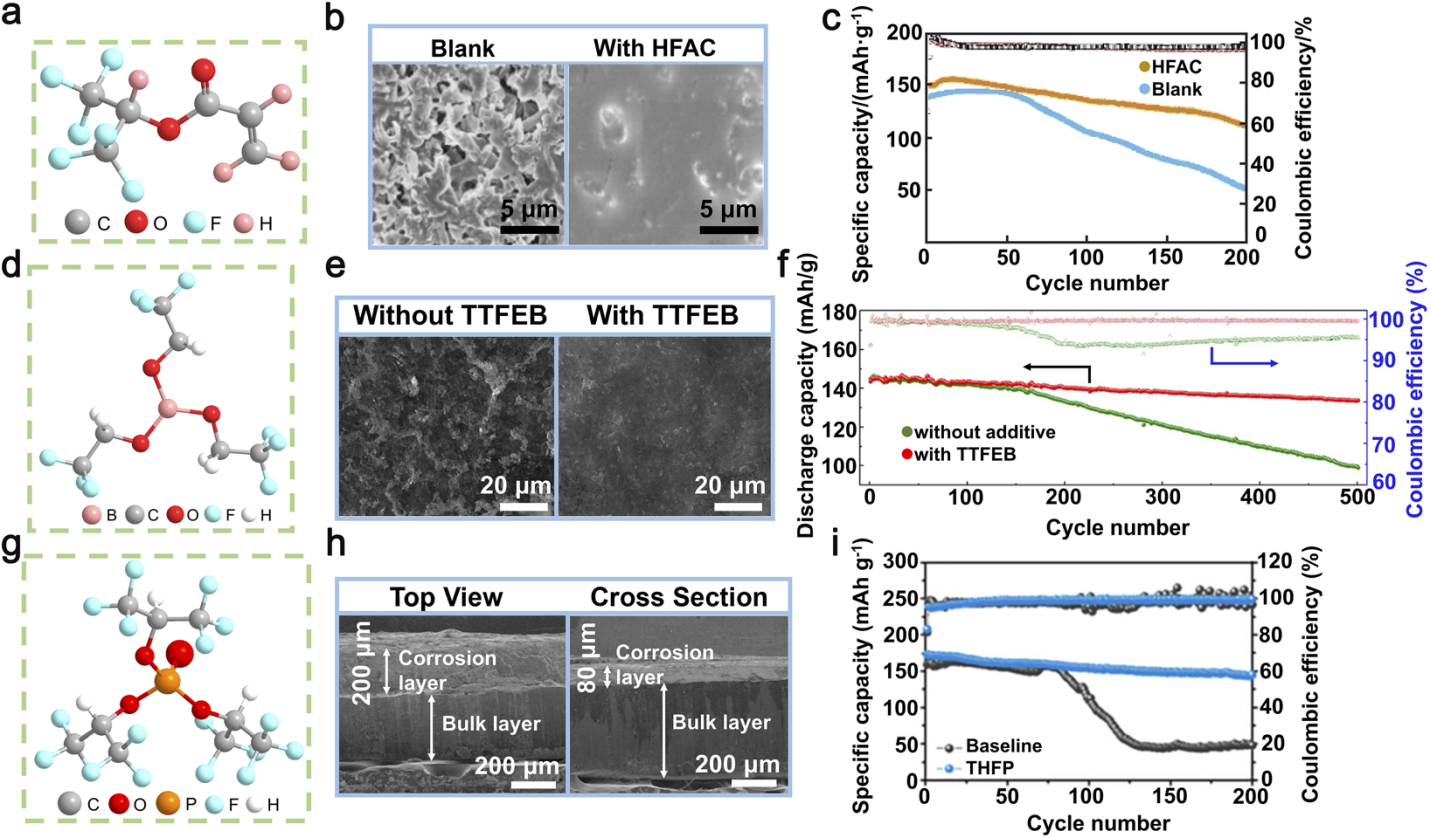
(a) The chemical structure of HFAC. (b) SEM images of Li anodes in the blank and 1.0 wt% HFAC-containing electrolytes after 10 cycles. (c) The cycling efficiency of Li‖NCM622 cells with and without the addition of 1.0 wt% HFAC. Reproduced with permission from ref. 101. Copyright 2022, Wiley-VCH. (d) The chemical structure of TTFEB. (e) SEM images depicting the morphology of lithium deposition on Li‖Cu batteries with/without TTFEB following 50 cycles. (f) The cycling efficiency of Li‖LiFePO_4_ full cells with/without TTFEB was evaluated at a 1C rate following three activation cycles conducted at a 0.1C rate. Reproduced with permission from ref. 102. Copyright 2017, Elsevier. (g) The chemical structure of THFP. (h) SEM image showcasing the formation of lithium deposits in symmetric Li‖Li cells containing 2.0 wt% THFP. (i) The charge–discharge behavior of Li‖NCM622 full cells, including THFP, was analyzed for the 200th cycle. Reproduced with permission from ref. 103. Copyright 2022, Elsevier.

During the preparation of LIBs, even minute amounts of water in the electrolyte can lead to irreversible damage to the negative metal electrode due to its rapid reaction with lithium metal. This reaction generates LiOH and Li_2_O on the surface, disrupting the composition of the SEI layer and potentially depleting valuable active materials.^121,122^ Additionally, in carbonate electrolytes, water molecules can cause the decomposition of LiPF_6_, leading to the production of corrosive HF and further damage the Li metal anode.^123,124^ In 2022, Li *et al.* proposed a hydrophobic solvation structure for Li-ions to enhance the cycling stability of LMBs by incorporating hexafluoroisopropyl acrylate (HFAC) as an electrolyte additive (Fig. 4a).^101^ The high dipole moment of the HFAC molecule, measuring 2.87 D, facilitates its coordination with Li-ions. This interaction alters the solvation structure, thereby increasing the proportion of PF_6_^−^ anions within the solvated complex from 0.66 to 0.77. In the molecular structure of HFAC, specific olefin groups and non-polar perfluorocarbon chains (–CF_2_CF_2_CF_3_) exhibit pronounced hydrophobicity. This characteristic promotes the formation of a hydrophobic layer around Li-ions during solvation. This layer with hydrophobic properties effectively hinders the chemical interaction between LiPF_6_ and minute quantities of water (H_2_O), thus minimizing the likelihood of producing decomposition by-products that can impede ion conductivity at both the lithium anode and cathode. Furthermore, HFAC facilitates the abundant formation of lithium-affinitive C–F bonds and a minor amount of LiF. These C–F bonds have the ability to rapidly adsorb and immobilize Li-ions, facilitating their even deposition on the surface of the electrode. This not only reduces the occurrence of lithium dendrites but also provides additional stability and safety in terms of electrochemical performance by further enhancing the SEI. After deposition–exfoliation for 10 cycles, as shown in scanning electron microscopy (SEM) images, the HFAC-modified Li metal anode can still maintain high integrity without any Li dendrites or dead Li, while its counterparts are covered with mossy Li dendrites and a lot of exfoliated Li (Fig. 4b). When applied in Li‖Li symmetric batteries, HFAC can support long-term deposition–stripping cycling for more than 500 hours at 1 mA cm^−2^. When utilized in NCM622‖Li full batteries, the inclusion of HFAC enhances the specific capacity of the full battery after 200 cycles to 111 mAh g^−1^, which accounts for approximately 74% of its initial capacity. This is notably higher compared to the full battery with a blank electrolyte (50.7 mAh g^−1^) as depicted in Fig. 4c.^56^

The movement of Li-ions within the electrolyte is crucial in the development and expansion of lithium dendrites.^125,126^ Studies have indicated that the arrangement of ions in close proximity to the positive electrode has a notable impact on both the structure and transference efficiency of Li-ions within the electrolytic solution. Enhancing the transport routes for Li-ions is regarded as a viable approach to alleviate ion concentration gradients, improve electrochemical efficiency, and regulate the uneven morphology of the lithium anode.^127–129^ While there have been several suggestions to enhance the transference numbers of Li-ions, such as employing single-ion electrolytes and “wet sand” electrolytes,^130,131^ the exploration of electrolyte additives for this purpose has been relatively limited. The inclusion of ester additives can contribute to the creation of a durable and conductive film known as the SEI. This SEI layer has the potential to significantly improve the movement of Li-ions, thereby augmenting the transference number of Li-ions. In 2017, Ma *et al.* presented TTFEB (tris(2,2,2-trifluoroethyl) borate), a dual-purpose electrolyte additive aimed at enhancing the efficiency of lithium anodes (Fig. 4d).^102^ With the addition of 2% TTFEB, the fluorine groups facilitate the formation of an SEI layer rich in LiF and enhance Li-ion migration within the electrolyte, resulting in uniform lithium deposition and effective suppression of dendrite growth (Fig. 4e). The addition of TTFEB additives to electrolytes significantly enhances the coulombic efficiency of Li‖Cu batteries (reaching approximately 96%) and prolongs the cycle life of Li‖Li batteries (exceeding 1000 hours), while simultaneously maintaining stable polarization and SEI resistance. The results of further investigations demonstrated that Li‖LiFePO_4_ full batteries incorporating 2% TTFEB exhibit a consistently high specific capacity of 133.6 mAh g^−1^ and an exceptional coulombic efficiency exceeding 99.5% even after undergoing 500 cycles at a rate of 0.1C (where 1C corresponds to a current density of 170 mA g^−1^) (Fig. 4f). This demonstrates the immense potential of bifunctional TTFEB additives in rechargeable LMBs. The surface properties of the lithium anode are not only modulated, but also the overall performance of the electrolyte is improved by these additives, offering novel insights into the multifaceted roles of electrolyte additives in rechargeable LMBs.^102^

The organic ester additives play a dual role in optimizing the migration of Li-ions within the electrolyte, thereby enhancing the overall ionic conductivity of the battery and stabilizing the structure of the lithium metal anode.^132,133^ In 2022, a pioneering investigation conducted by Sun *et al.* unveiled a revolutionary electrolyte that utilizes the non-combustible compound triethyl phosphate (TEP) as its base, supplemented with tris(hexafluoroisopropyl) phosphate (THFP) as a crucial additive (Fig. 4g).^103^ The unique chemical properties of the THFP additive can be attributed to its polar C–F bonds and the presence of numerous CF_3_ groups, which result in significantly lower LUMO (Lowest Unoccupied Molecular Orbital) and HOMO (Highest Occupied Molecular Orbital) energies. This facilitates the formation of a structurally stable, LiF-rich SEI layer through the reductive reactions involving THFP. Secondly, the structural design of THFP contributes to its increased affinity for PF_6_^−^ anions, effectively inhibiting the formation of lithium dendrites (Fig. 4h) and reducing electrolyte decomposition. For symmetric Li‖Li batteries and Li‖NCM622 full batteries, the addition of THFP resulted in two notable enhancements. First, the degree of polarization in the batteries was reduced, leading to better electrochemical stability. Second, there was a significant increase in the longevity of the batteries. Specifically, when subjected to 200 cycles at a current density of 100 mA g^−1^, the Li‖NCM622 full battery exhibited a specific capacity of 145 mAh g^−1^ (Fig. 4i).^103^

**Fig. 5:**
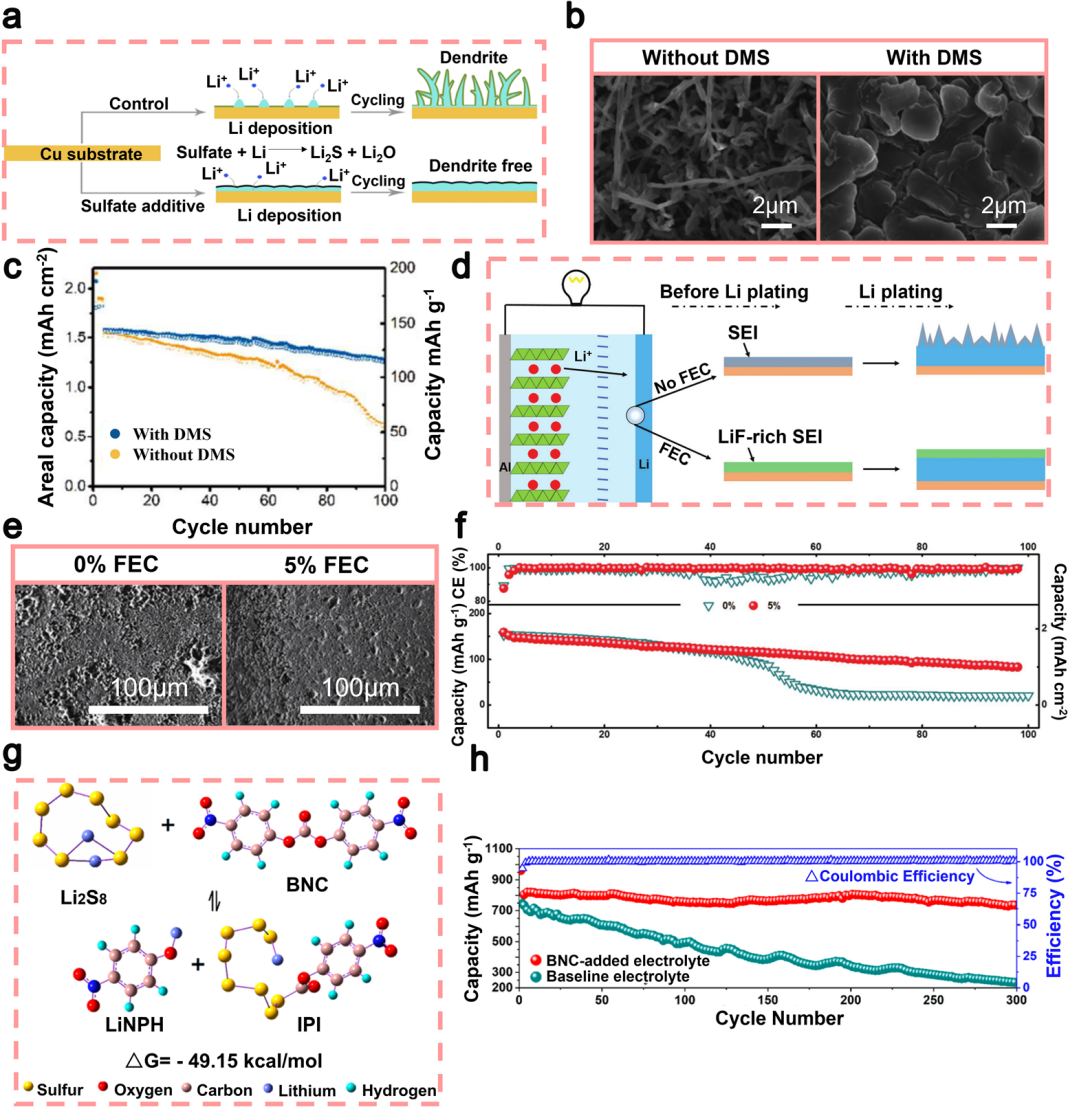
(a) Schematic illustrations depicting the deposition of Li in electrolytes, with or without DMS. (b) SEM images were obtained to observe the initial deposition of Li in electrolytes under different conditions: without/with dimethyl sulfoxide (DMS), at a current density of 0.5 mA cm^−2^. (c) The performance of Li‖NCM batteries in cycling. Reproduced with permission from ref. 104. Copyright 2017, American Chemical Society. (d) Schematic diagrams showing the impact of the FEC additive on the lithium metal anode. (e) SEM was used to examine the morphology of Li deposition on Cu foils before and after 50 cycles, both with and without the addition of 5% FEC. (f) The cycling efficiency of Li‖NMC batteries with 0% and 5% FEC, operating at a rate of 1.0C (equivalent to a current density of 180 mA g^−1^), was evaluated after one initial activation cycle performed at a lower rate of 0.1C. Reproduced with permission from ref. 105. Copyright 2017, Wiley-VCH. (g) The complexes of Li_2_S_8_ with insoluble sulfur compounds BNC and 4-nitrophenolate lithium. (h) The performance of Li–S batteries incorporating BNC for more than 300 cycles of cycling. Reproduced with permission from ref. 106. Copyright 2019, American Chemical Society.

Ester additives exhibit remarkable chemical stability and play a pivotal role in forming a consistent and stable SEI layer. The SEI layer functions as a highly efficient barrier separating lithium metal from the electrolyte, thereby minimizing unnecessary side reactions and consequently extending the lifespan of batteries. In 2018, Wan *et al.* successfully utilized dimethyl sulfate (DMS, C_2_H_6_SO_4_) as an additive to facilitate the formation of a robust SEI layer.^104^ The preferential reduction of DMS resulted in the formation of a lithium surface with an SEI layer composed of Li_2_S/Li_2_O (Fig. 5a). The high structural modulus and low interfacial resistance of this inorganic SEI layer facilitate dense and dendrite-free lithium deposition (Fig. 5b). In addition, the presence of this layer serves to hinder direct interaction between deposited lithium and corrosive electrolytes, thereby augmenting the durability of the lithium anode over multiple cycles. Surprisingly, the coulombic efficiency reached an impressive average of 97%, while the cycle life was extended to 150 cycles. The utilization of DMS additives in Li‖NCM full batteries also led to notable enhancements in the longevity of LMBs. After 100 cycles, the capacity retention rate increased from 62% to 85% (Fig. 5c).^59^ In 2016, Zhang *et al.* utilized FEC as an additive to create an SEI layer enriched with LiF (Fig. 5d).^105^ The SEI layer formed by FEC promotes the uniform deposition of lithium, preventing the formation of lithium dendrites due to its compact and stable nature (Fig. 5e). Consequently, the Li‖Cu half-batteries exhibited a remarkable enhancement in coulombic efficiency, reaching an impressive value of 98% after undergoing 100 cycles. When combining the lithium metal anode, which is protected by FEC, with a high-loading Ni_0.5_Co_0.2_Mn_0.3_O_2_ (NCM) cathode, we achieved an impressive initial capacity of 154 mAh g^−1^ (equivalent to 1.9 mAh cm^−2^) when operating at a rate of 1C (where 1C = 180.0 mA g^−1^). Furthermore, even after 100 cycles, the battery retained approximately 65% of its original capacity (Fig. 5f).^105^

Extensive research has been conducted on Li–S batteries as a solution for storing large amounts of energy. However, the practical application of these batteries faces significant obstacles due to the difficulties posed by lithium anodes and organic liquid electrolytes.^134,135^ One crucial concern pertains to the disintegration and transportation of Li_2_S_*x*_ intermediates throughout the discharge process, leading to uneven sedimentation and the formation of lithium dendrites.^136,137^ Ester additives can alter the microscopic structure of the electrolyte and thereby inhibit dendrite formation, enhancing battery performance. In 2019, Yang *et al.* sought to address these concerns by incorporating bis(4-nitrophenyl) carbonate (BNC) as an additive in the liquid electrolyte.^106^ BNC reacts with soluble Li_2_S_*x*_ polysulfides to form insoluble sulfide complexes and 4-nitrophenolate lithium (Fig. 5g). These insoluble sulfide complexes prevent the shuttling of soluble polysulfides from the conductive cathode to the anode surface, while the lithium 4-nitrophenolate (LNPH) by-product forms a dense passivating SEI layer on the anode, effectively inhibiting dendrite growth. Moreover, it exhibits high Li-ion conductivity, enabling low-impedance, rapid, and safe plating/stripping of lithium metal. Li–S batteries, when combined with the BNC additive, demonstrated an impressive capacity retention of 778.7 mAh g^−1^ even after undergoing more than 300 cycles at a rate of 1C. Additionally, the decay rate for each cycle was only 0.025%, and the coulombic efficiency approached nearly perfect levels (Fig. 5h).^106^ The BNC additive provides a cost-effective and secure solution for Li–S batteries, endowing lithium metal anodes with extended cycle life, which is of significant importance in energy storage.

**Fig. 6:**
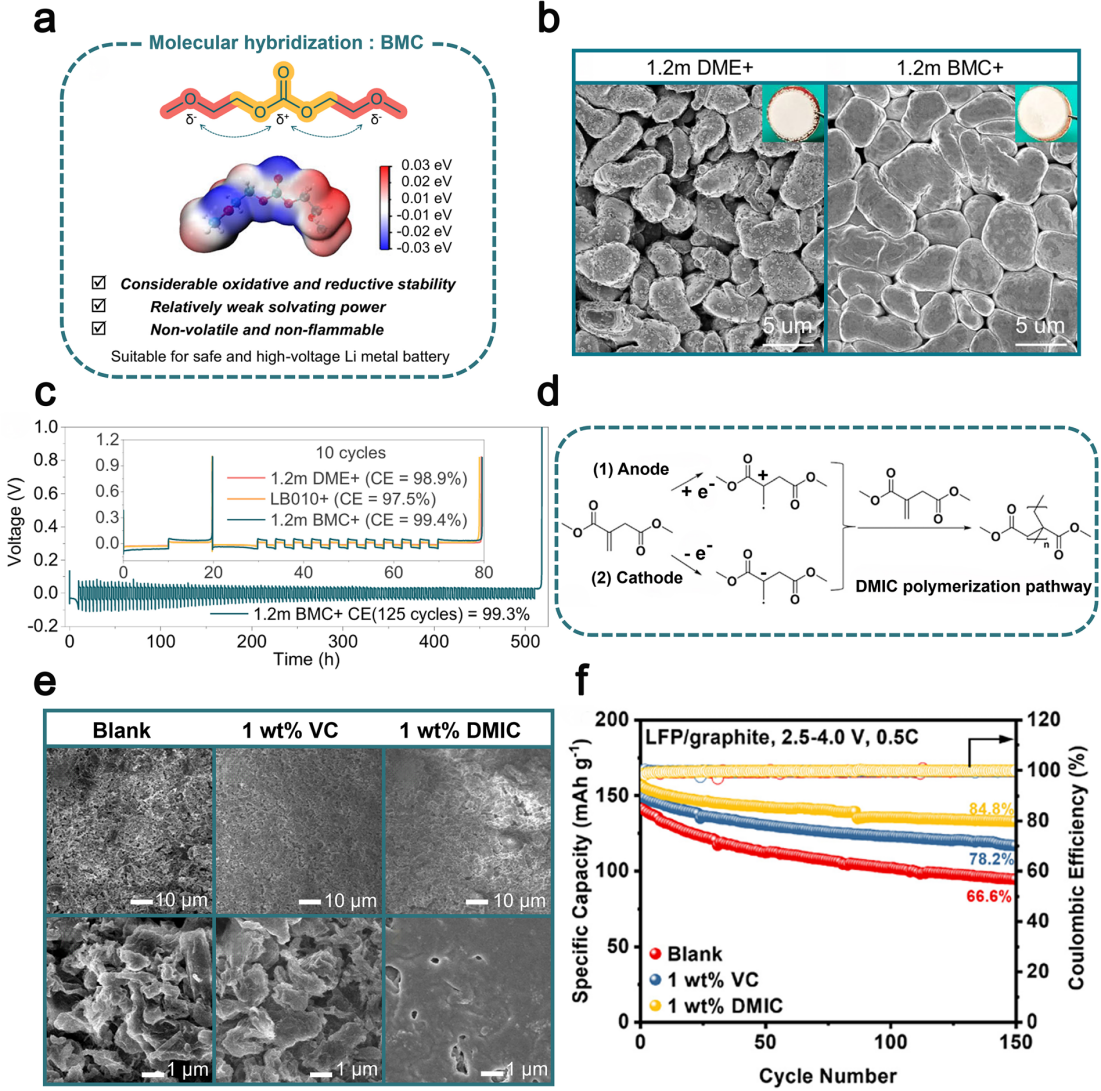
(a) Molecular structures, electrostatic potential (ESP) maps and characteristics of well-designed BMC solvent. (b) SEM and optical images for the surface of Li electrodeposits after the first plating on the Cu foil at 0.5 mA cm^−2^ and 3 mAh cm^−2^ in 1.2 m DME^+^ and 1.2 m BMC^+^. (c) Long-term Li plating/stripping CE test performed at 0.5 mA cm^−2^ and 1 mAh cm^−2^ in 1.2 m BMC^+^ (the inset shows the results obtained within 10 cycles in various electrolytes). Reproduced with permission from ref. 107. Copyright 2022, Springer Nature. (d) Possible mechanism of oxidation/reduction induced reaction of DMIC on the surface of the anode and cathode. (e) SEM images of the SEI on the Li metal surface after 500 cycles based on blank electrolyte, electrolyte containing 1 wt% VC, and electrolyte containing 1 wt% DMIC. (f) Long-term cycling performance of LFP/graphite pouch-batteries at 0.5C. Reproduced with permission from ref. 108. Copyright 2025, Royal Society of Chemistry.

In 2024, Chen *et al.* introduced bis(2-methoxyethyl) carbonate (BMC), a novel ester additive featuring a hybrid molecular design that combines ether and carbonate functionalities within a single molecule (Fig. 6a).^107^ This structural hybridization effectively balances electron distribution, endowing BMC with improved oxidative and reductive stability, as well as relatively weak solvation power.^138–140^ When applied as a single-solvent electrolyte for LMBs, the optimized BMC-based electrolyte demonstrated impressive performance characteristics, including a high coulombic efficiency of 99.4% during Li plating/stripping cycles (Fig. 6b and c). This electrolyte also exhibited excellent high-voltage tolerance up to 4.4 V, enabling outstanding cycling stability in practical Li-metal full batteries with high-loading NCM811 cathodes. Specifically, Li‖NCM811 full batteries retained 92% of their initial capacity after 150 cycles under demanding conditions (high cathode loading of 4.8 mAh cm^−2^). Additionally, the use of BMC significantly enhanced thermal and mechanical safety, as demonstrated by pouch batteries passing rigorous nail penetration tests and achieving improved thermal stability up to 155 °C. The molecular-level integration strategy proposed in this study provides a valuable pathway for designing advanced electrolytes that simultaneously meet stringent requirements for high energy density and safety in next-generation LMBs.

In 2025, Wang *et al.* presented bio-derived dimethyl itaconate (DMIC) as a sustainable, cost-effective electrolyte additive, synthesized from renewable itaconic acid using *Aspergillus terreus* fermentation (Fig. 6d).^108^ The distinctive α,β-unsaturated ester structure of DMIC facilitates dual interfacial stabilization by preferentially undergoing reduction at the anode (LUMO: −1.72 eV) and oxidation at the cathode (HOMO: −7.76 eV), forming robust and gradient organic–inorganic SEI/cathode electrolyte interphase (CEI) layers (Fig. 6e). The incorporation of DMIC notably reduces side reactions between electrolyte solvents and electrode surfaces, enhances Li-ion transport kinetics, and significantly lowers internal resistance.^141–143^ As a result, DMIC-containing electrolytes demonstrated superior cycling stability, rate capability, and extended performance lifetime. Specifically, LiFePO_4_‖Li batteries exhibited a notable capacity retention of 103.1 mAh g^−1^ even at a high discharge rate of 4C. Meanwhile, LiNi_0.6_Co_0.2_Mn_0.2_O_2_ (NCM_622_)‖Li batteries employing DMIC achieved significantly improved cycling stability, retaining 76.5% capacity after 500 cycles at 2C, surpassing conventional VC-based electrolytes. Additionally, DMIC showcased promising practicality in pouch batteries (LFP‖graphite), maintaining an impressive 84.8% capacity retention after 150 cycles at 0.5C (Fig. 6f). This performance, combined with DMIC's lower production cost and environmentally friendly synthesis, underscores its substantial potential as an advanced, green electrolyte additive for lithium metal battery technologies.

Incorporating ester-based additives into the electrolyte of LMBs presents numerous benefits. (i) The inclusion of ester additives aids in the development of a steadfast and water-repellent SEI layer. For example, HFAC has a high dipole moment, allowing it to coordinate with Li-ions, thereby enhancing the stability and hydrophobicity of the SEI. Similarly, BMC integrates carbonate and ether functionalities, forming a stable and hydrophobic SEI layer that enhances battery safety and longevity. (ii) Ester additives can inhibit dendritic crystal growth. For example, the utilization of THFP results in a reduction of energy levels for both the LUMO and HOMO, facilitating the development of an SEI layer rich in LiF. This effectively inhibits dendrite proliferation. DMIC also contributes significantly to dendrite suppression through its robust organic–inorganic SEI formation at the lithium anode. (iii) Ester additives improve ionic conductivity. For example, TTFEB promotes the formation of an SEI membrane enriched in LiF and enhances lithium-ion migration in the electrolyte, resulting in uniform lithium deposition and effective dendrite suppression. (iv) Ester additives enhance coulombic efficiency. The integration of DMS forms a composited SEI layer with low interfacial resistance, achieving coulombic efficiencies of up to 97%. (v) Ester additives can alter the microscopic structure of the electrolyte to improve battery performance. For instance, the interaction between BNC and soluble Li_2_S_*x*_ polysulfides results in the formation of sulfur complexes that are not soluble. This effectively hinders the movement of polysulfides and addresses concerns associated with uneven deposition and dendrite growth in Li–S battery systems. However, there are challenges in using ester-based additives. While they show good performance in stabilizing the SEI layer, they may interfere with cathode materials, requiring consideration of the compatibility of ester additives. Additionally, although ester additives improve the performance of lithium metal under laboratory conditions, whether they can maintain the performance and safety of LMBs when transitioning to industrial applications remains an open question.

### 3.2. Organic sulfide additives

Organic sulfide additives often demonstrate superior effectiveness in ether-based electrolytes, particularly in Li–S battery systems, owing to their ability to modulate lithium-ion solvation and polysulfide interaction. In recent years, organic sulfides have increasingly garnered attention as electrolyte additives to enhance the efficiency of LMBs.^144,145^ The utilization of organic sulfide additives can enhance the cycle stability and safety of batteries, prolong the lifespan of LMBs, and elevate their performance, all while exerting minimal impact on the internal battery environment.^146,147^

**Fig. 7:**
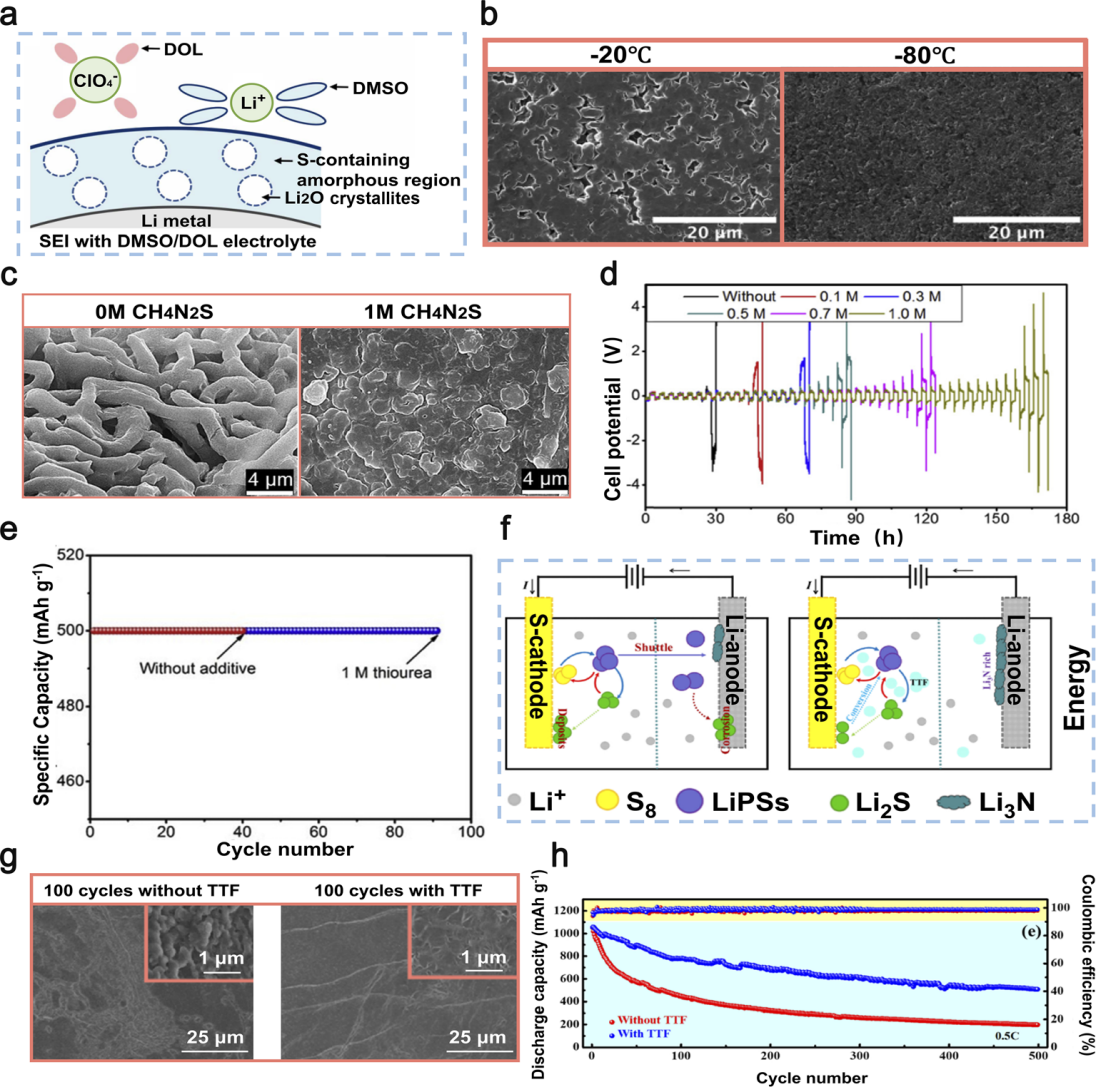
(a) Schematics of the Li-ion solvation behavior and SEI structure in DMSO/DOL electrolyte. (b) The SEM images depict the lithium deposition in an electrolyte containing DMSO/DOL at temperatures of −20, and −80 °C. Reproduced with permission from ref. 109. Copyright 2022, Royal Society of Chemistry. (c) The SEM images depict the Li deposition in Li‖Cu batteries after electroplating, with and without the addition of CH_4_N_2_S. (d) The cycling performance of symmetric batteries was evaluated in electrolytes with varying concentrations of CH_4_N_2_S (0–1 M). (e) Performance of Li–O_2_ batteries in terms of charge–discharge cycling under constant current conditions, with and without CH_4_N_2_S in the electrolyte. Reproduced with permission from ref. 110. Copyright 2018, Elsevier. (f) Schematic of the cycling mechanism of Li–S batteries with TTF as an additive. (g) SEM images of the surface of the lithium anode in Li–S batteries with/without TTF after 100 cycles. (h) Long-term cycling capability graph of Li–S batteries with/without TTF at 0.5C. Reproduced with permission from ref. 111. Copyright 2022, Elsevier.

One of the primary obstacles encountered in the advancement of energy storage technologies, such as batteries, lies in their capacity to function optimally under frigid conditions. One significant obstacle to the low-temperature application of LMBs is the occurrence of unavoidable battery failures at sub-zero temperatures. Recently, there has been some progress in improving the performance of LMBs in harsh environments through the use of organic sulfides as electrolyte additives. In 2022, Zhang *et al.* introduced dimethyl sulfoxide (DMSO) as an additive in a 1,2-dichloroethane (DOL)-based electrolyte.^109^ DMSO exerts a significant influence on the solvation sheath of Li-ions in the DMSO/DOL electrolyte, thereby impacting the behavior of Li deposition. This results in the development of a strong SEI layer on the lithium metal electrode at extremely low temperatures (Fig. 7a). Through SEM, Zhang *et al.* observed lithium deposition at different temperatures and found that as the temperature of the electrolyte containing DMSO decreases, the lithium deposition becomes more compact (Fig. 7b). In addition, Li‖LiFePO_4_ full batteries with DMSO electrolyte exhibited discharge capacities of 107 mAh g^−1^ and 51 mAh g^−1^ at a rate of 0.2C, under temperature conditions of 0 °C and −40 °C respectively.^109^ The presence of DMSO enables the battery to sustain a favorable capacity.

Organic sulfide additives can inhibit the rapid degradation of beneficial components in the electrolyte during cycling, thereby enhancing the efficacy of dendrite suppression on the Li anode.^148,149^ In 2018, Ho *et al.* employed thiourea (CH_4_N_2_S) as an electrolyte additive for LMBs.^110^ The utilization of thiourea as an additive in copper plating solutions is well-documented. During the plating process, sulfur atoms from thiourea are adsorbed onto the electrode surface, thereby exerting an influence on the formation and distribution of copper deposits. When CH_4_N_2_S is added to LiTFSI/TEGDME electrolytes, it minimizes the decomposition of the LiTFSI salt, reaching the establishment of a consistent and steady SEI layer on the Li electrode. This SEI layer effectively inhibits further electrolyte decomposition, thus curtailing the growth of Li dendrites (Fig. 7c). Rechargeable lithium batteries with the addition of 1.0 M CH_4_N_2_S showed a significant improvement in their cycling lifespan, achieving 43 cycles at a current density of 1 mA cm^−2^, which is six times higher compared to those without the additive (Fig. 7d). Furthermore, Ho *et al.* successfully applied the CH_4_N_2_S additive to lithium–oxygen batteries to improve their cyclability. The Li–O_2_ batteries with CH_4_N_2_S exhibited a significantly enhanced cycle life of 90 cycles (Fig. 7e) at a constant capacity of 500 mAh g^−1^, surpassing that of batteries without the additive by more than twofold.^110^ Thiourea as an additive has a notable impact in enhancing both the cyclability and coulombic efficiency of Li–O_2_ batteries.

Organic sulfides effectively stabilize the SEI layer on lithium metal anodes, thereby mitigating the risk of lithium dendrite formation. In 2022, Wu *et al.* led a research team that employed tetrathiafulvalene (TTF) as an electrolyte additive in Li–S batteries to achieve superior battery performance.^111^ The presence of TTF, acting as a π-electron donor, enhances the electron transport characteristics in Li_2_S_*x*_, which are influenced by van der Waals interactions. Additionally, it facilitates the formation of a highly conductive passivation layer on the lithium metal anode composed of LiNO_3_ and Li_3_N (Fig. 7f). After 100 cycles, the lithium anode surface in batteries incorporating TTF maintains an exceptionally smooth texture. This is mainly attributed to the capability of TTF to expedite the transformation process of lithium polysulfides (LiPSs), consequently impeding the erosion of the lithium anode and guaranteeing the preservation of the Li–S anode surface's integrity (Fig. 7g). When subjected to a current density of 0.1C, these batteries exhibit a discharge capacity reaching as high as 1359 mAh g^−1^. In addition, even after undergoing 500 cycles (Fig. 7h), the battery exhibits a consistent specific capacity of 509 mAh g^−1^ when subjected to a current density of 0.5C.^111^ In general, the addition of TTF as an electrolyte supplement effectively inhibits undesired interactions between the lithium anode and LiPSs, decreases electrolyte deterioration, and significantly improves the long-term performance of Li–S batteries. This pivotal contribution lays a solid foundation for future advancements in Li–S battery technology.

Organic sulfides offer numerous advantages as electrolyte additives for LMBs. Firstly, in DMSO/DOL electrolytes, DMSO as an organic sulfide predominantly governs the solvation of Li-ions, influencing their deposition behavior and thereby facilitating the development of a robust SEI layer. Particularly under low-temperature conditions, DMSO enables more compact lithium deposition. Secondly, CH_4_N_2_S decelerates the rapid degradation of beneficial components within the electrolyte during cycling. It achieves this by adsorbing sulfur atoms onto the electrode surface, effectively suppressing the proliferation of lithium dendrites. Thiourea has been effectively utilized to improve the cycling efficiency of Li–O_2_ batteries. Thirdly, the utilization of TTF effectively minimizes undesired interactions between the lithium anode and LiPSs, resulting in reduced degradation of the electrolyte and a remarkable enhancement in the battery's cycling stability. However, the use of organic sulfides as additives also presents several challenges. First, these additives may react adversely with the electrolyte or other battery components, necessitating further research to elucidate these interactions. Second, although DMSO performs well at low temperatures, organic sulfides generally lose their stability or efficacy under extreme temperature or pressure conditions. The role of organic sulfides in LMBs under such extreme conditions remains to be explored. Third, the use of high-purity or specialized organic sulfide additives may increase the manufacturing costs of batteries. Fourth, the long-term impact of organic sulfides on the battery's stability and reliability has not been fully understood, requiring additional long-term studies. In summary, the application of DMSO results in the formation of a strong SEI layer on the lithium metal electrode even in low temperature environments. This finding offers a potential solution to the challenges faced by LMBs when operating at sub-zero temperatures. CH_4_N_2_S reduces the degradation of LiTFSI salts, creates a consistent and enduring SEI layer on the Li electrode, hampers the proliferation of Li dendrites, and enhances the longevity of Li–O_2_ batteries. The utilization rate of sulfur in Li–S batteries is significantly improved by TTF, while also effectively preventing the shuttle effect. Additionally, TTF facilitates the development of a highly conductive passivation layer between LiNO_3_ and Li_3_N on the lithium anode, thereby impeding the growth of lithium dendrites. It can be seen that organic sulfides offer great benefits for LMBs, Li–S batteries and Li–O_2_ batteries by stabilizing the SEI and improving performance.

### 3.3. Carboxylic acid compound additives

Carboxylic acid compound additives are predominantly employed in carbonate-based electrolytes, leveraging their ability to chemically interact with lithium metal surfaces and facilitate the formation of stable, dense SEI layers in such polar environments. Carboxylic acid compounds play various crucial functions as electrolyte additives in LMBs, such as augmenting the durability of the SEI layer, inhibiting the proliferation of lithium dendrites, enhancing the battery's cycling efficiency, and elevating its electrochemical stability and safety. These functions not only enhance the overall battery performance, but also provide a viable solution to tackle the durability and safety concerns linked with LMBs.

**Fig. 8:**
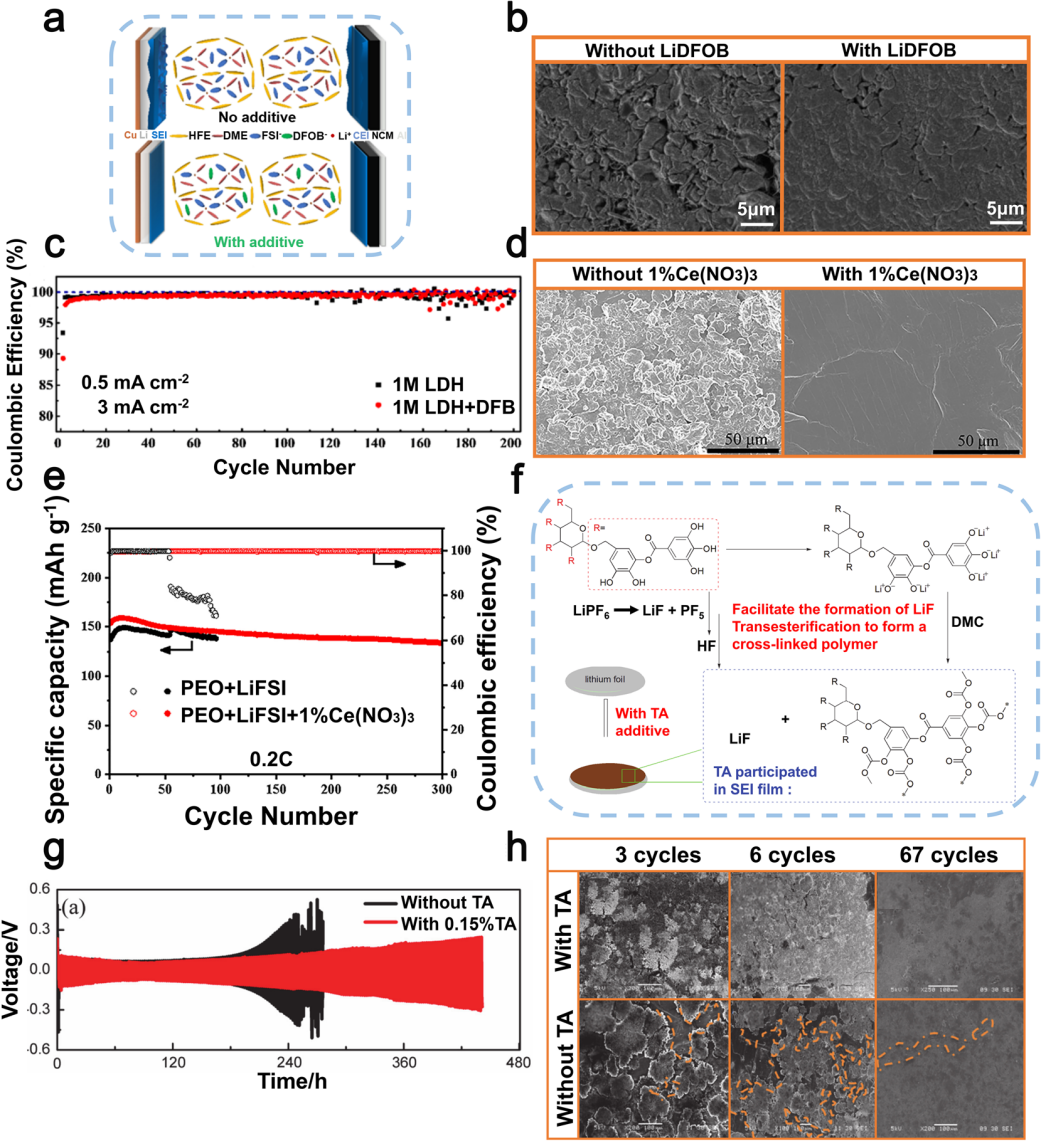
(a) Schematic diagrams of anodes and cathodes in electrolytes with two different solvation structures. (b) SEM images depicting the occurrence of lithium plating in LDH electrolyte under two different conditions: without LiDFOB and with LiDFOB, both at a current density of 1 mAh cm^−2^. (c) coulombic efficiency of Li‖Cu batteries. Reproduced with permission from ref. 112. Copyright 2022, Elsevier. (d) SEM images of the surface morphology of lithium metal anodes in Li|PEO + LiFSI|Li batteries, both with and without the addition of Ce(NO_3_)_3_, were obtained after 50 cycles. (e) Cycling performance of Li‖FePO_4_ full batteries in PEO + LiFSI electrolyte, with and without the addition of 1% Ce(NO_3_)_3_. Reproduced with permission from ref. 113. Copyright 2021, Elsevier. (f) Mechanism of formation of organic/inorganic composite SEI layers on the lithium metal anode with film-forming additive TA. (g) The cycling performance of Li‖Li symmetric batteries with/without the addition of TA. (h) The planar SEM images of Li‖Li symmetric batteries with/without the addition of TA after various cycles. Reproduced with permission from ref. 114. Copyright 2020, Elsevier.

In 2023, Zhang *et al.* cleverly introduced a minute quantity of LiDFOB as an electrolyte additive at a concentration of 0.02 M.^112^ This alteration resulted in an increase in the oxidative stability of the electrolyte up to 4.3 V, while also significantly improving the efficiency of both lithium metal electroplating and stripping processes, leading to a notable enhancement in coulombic efficiency. In the anode region, LiDFOB efficiently promoted the complete breakdown of FSI^−^ anions, resulting in the development of a strong SEI layer that contains high concentrations of Li_3_N and LiF (Fig. 8a). This particular attribute ensured more even and compact lithium deposition on the electrode (Fig. 8b), resulting in improved uniformity and density. In the presence of LiDFOB, a uniform and dense CEI layer was formed on the NCM811 material surface, effectively mitigating cathode surface reactivity and minimizing transition metal dissolution. Upon subjecting the Li‖Cu batteries containing the LiDFOB additive to 200 cycles, a remarkable coulombic efficiency of up to 99.4% was achieved (Fig. 8c) while operating at a current density of 0.5 mA cm^−2^ and exhibiting a lithium plating capacity of 3 mAh cm^−2^.^112^ In summary, LiDFOB functions as a beneficial electrolyte additive that enhances the durability and consistency of the SEI layer on the lithium metal anode while also achieving exceptional coulombic efficiency. This provides valuable insights for researching carboxylate compounds as electrolyte additives in LMBs.

In 2022, Yang *et al.* achieved a significant breakthrough by creating a flexible all-solid-state electrolyte using Ce(NO_3_)_3_, lithium bis(fluorosulfonyl)imide (LiFSI), and polyethylene oxide (PEO).^113^ The addition of Ce(NO_3_)_3_ proved to be beneficial, promoting the creation of a consistent and enduring SEI on the lithium metal anode. This skillfully engineered SEI assists in regulating the movement of Li-ions, effectively reducing dendritic growth and improving the safety of the lithium anode (Fig. 8d). Furthermore, this formulation also significantly improved the ionic conductivity of the SPEs. The experimental findings demonstrated that, at a temperature of 60 °C, the electrolyte displayed an exceptional ionic conductivity of 1.6 × 10^−4^ S cm^−1^. Additionally, it exhibited a wide electrochemical stability range of 4.4 V. When utilized in Li‖Li symmetric batteries at 60 °C and 0.1 mA cm^−2^, the cycle life exceeded 1100 hours. In addition, LiFePO_4_‖Li batteries fabricated using this electrolyte exhibited an initial specific capacity of 153.7 mAh g^−1^ and retained 87% of this capacity (133.7 mAh g^−1^) after undergoing 300 cycles at a rate of 0.2C, while maintaining a coulombic efficiency of 99.8% (Fig. 8e).^113^ In general, the utilization of Ce(NO_3_)_3_ as a highly effective component in the electrolyte significantly improves both the conductivity of ions and interfacial stability, thereby facilitating the practical implementation of LMBs.

Tannic acid (TA), a cost-effective and abundantly available polyphenolic compound, has garnered widespread attention in the material engineering of LIBs. In 2020, a study by Ran *et al.* affirmed its efficacy as a high-performance additive for safeguarding the lithium metal anode.^114^ Including small quantities of TA in the electrolyte base solution consisting of 1 mol L^−1^ LiPF_6_^−^ in EC/DMC/EMC (1 : 1 : 1) facilitates the catalytic hydrolysis process, resulting in the formation of LiF from LiPF_6_. Subsequent polymer products crosslink with DMC through ester exchange reactions, giving rise to a homogeneous and stable organic/inorganic composite solid-electrolyte interphase (SEI) film (Fig. 8f). According to empirical data, the inclusion of 0.15 wt% TA resulted in an enhancement in the cycling stability of symmetric Li‖Li batteries. Specifically, at a current density of 1 mA cm^−2^ and a capacity of 1 mAh cm^−2^ (Fig. 8g), the duration before failure increased from 170 hours to 270 hours. This enhancement is attributed to the inhibition of lithium dendrite growth (Fig. 8h), as well as an increase in the electrochemical efficiency of lithium metal.^114^ In summary, TA holds substantial promise as an effective protective additive for LMBs, offering significant advancements in both cycling stability and electrochemical performance.

Carboxylic acid compounds offer multifaceted advantages as electrolyte additives in LMBs. First and foremost, these compounds can form stable chemical bonds with Li-ions, optimizing the structure of the SEI layer to make it more uniform and stable. Secondly, the incorporation of additives aids in maintaining a well-balanced electric field distribution across the lithium metal surface, effectively impeding the growth of lithium dendrites and consequently improving the safety characteristics of the battery. The synergistic effects of these two elements dramatically improve the battery's cycle life. These three additives effectively highlight the significant advantages of carboxylic acid compounds as electrolyte additives in enhancing the performance of LMBs. LMBs with carboxylic acid additives demonstrate higher ionic conductivity, lower internal resistance, and superior coulombic efficiency during cycling.

### 3.4. Other small molecule additives

**Fig. 9:**
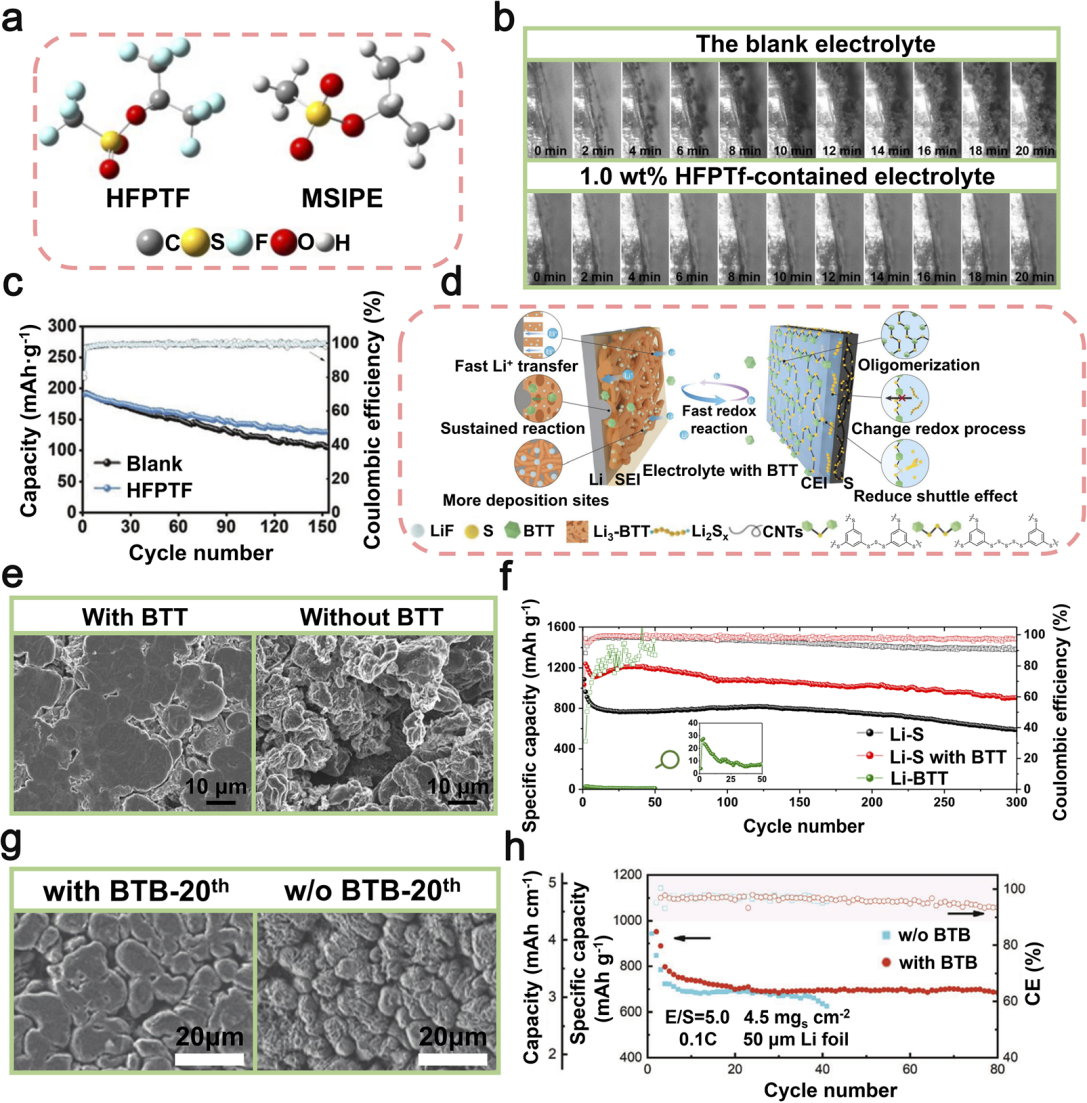
(a) Chemical structures of EC and DMC. (b) *In situ* optical microscopy examination of Li plating in a blank electrolyte and an electrolyte with 1.0 wt% HFPTF. (c) The cycling performance of Li‖Li symmetric batteries was evaluated in electrolytes without HFPTF, with a current density of 0.5 mA cm^−2^ and a capacity of 0.25 mAh cm^−2^; the cycling performance of Li‖Li symmetric batteries was studied using HFPTF-containing electrolyte at different concentrations, namely 0.5 wt%, 1.0 wt%, and 2.0 wt%. Reproduced with permission from ref. 115. Copyright 2021, Wiley-VCH. (d) Schematic diagram of a lithium battery with BTT-containing electrolyte, illustrating the formation of D-SEIs at the interfaces of the anode and cathode. (e) SEM images captured from a bird's-eye view depicting lithium foil within lithium batteries utilizing electrolytes containing BTT and those without, following 50 cycles at a rate of 0.5C. (f) Assessment of the cycling durability and coulombic efficiency of Li–S batteries with/without BTT at a 1C rate for over 300 cycles. Reproduced with permission from ref. 116. Copyright 2021, Springer Nature. (g) SEM images of lithium anodes in lithium batteries with/without BTB after the 20th cycle. (h) The performance of a lithium battery during cycling was evaluated under challenging conditions, including a high-load S cathode (4.5 mg cm^−2^), low E/S ratio (5.0 μL mg^−1^), and an ultra-thin lithium anode (50 μm) at 0.1C. Reproduced with permission from ref. 117. Copyright 2020, Wiley-VCH.

Other small molecule additives, such as sulfonic acid-based compounds, alcohol derivatives, and nitrile-containing molecules, exhibit versatile applicability across diverse electrolyte solvent systems, including carbonate-based and ether-based electrolytes, due to their unique functional group chemistry and excellent solubility. Sulfonic acid-based compounds play an indispensable role in LMBs due to their unique chemical properties. Specifically, these compounds exhibit excellent solubility and high ionic conductivity in electrolytes, facilitating uniform lithium-ion migration between electrolyte and the lithium metal anode, thus mitigating the proliferation of lithium dendrites. This can be mainly attributed to certain functional groups, such as –SO_3_H, which exhibit a strong interaction with Li-ions, resulting in the formation of a more uniform and stable SEI. In 2021, researchers led by Li utilized hexafluoroisopropyl trifluoromethanesulfonate (HFPTF) as an electrolyte additive to further enhance the performance of LMBs (Fig. 9a).^115^ HFPTF modifies the primary solvation arrangement of Li-ions in the electrolyte, facilitating their reduction to metallic lithium and uniform deposition on the electrode surface. Moreover, HFPTF facilitates the formation of a gradient Li_2_SO_*x*_ and a homogeneous LiF-type SEI at the lithium metal anode, as well as a uniform CEI at the cathode, thereby enhancing the overall stability of the electrode/electrolyte interface. This additive exhibits a significantly elevated reduction potential of 1.73 V, enabling its early involvement in the generation process of the SEI layer (Fig. 9b). The tests conducted on Li‖Li symmetric batteries, using both blank and HFPTF-containing electrolytes, demonstrated that the cycle stability could reach up to 1000 hours at a current density of 0.5 mA cm^−2^. Optimal performance was observed when the HFPTF content was maintained at 1 wt%. The incorporation of HFPTF as an electrolyte additive in a full-battery Li‖NMC811 configuration significantly enhanced the electrochemical performance of the battery. It demonstrated an impressive initial capacity of 190.1 mAh g^−1^ and maintained a capacity of 129.5 mAh g^−1^ even after undergoing 150 cycles at a rate of 100 mA g^−1^ (Fig. 9c).^115^ Overall, the addition of HFPTF not only enhances the effective formation of the SEI layer but also significantly contributes to the commercialization prospects of LMBs.

Expanding on our previous discussion regarding the improvement of sulfur utilization and the reduction of the shuttle effect in Li–S batteries by incorporating TTF additives, it is worth mentioning that alcohol-based additives such as BTT (1,3,5-benzenetrithiol) can also play a role in stabilizing the interface between the lithium metal anode and restraining the migration of lithium polysulfides (LiPSs). In 2021, a team led by Guo introduced an electrolyte additive known as BTT into the Li–S battery system.^116^ The addition of this compound facilitates the *in situ* formation of highly stable SEIs, or dynamic SEIs (D-SEIs), at both electrodes (Fig. 9d). The highly reactive sulfur–hydrogen (S–H) groups in BTT readily undergo reactions with lithium and sulfur, resulting in the formation of interface layers composed of S–Li and S–S bonds. Specifically, the formation of S–Li bonds mitigates lithium dendrite growth (Fig. 9e), enhancing the conductivity of Li-ions and enabling reversible lithium deposition and stripping. On the other hand, the presence of SEI layers containing S–S bonds directs the oligomerization process of sulfur, altering its redox pathway and effectively inhibiting the occurrence of the sulfur shuttle phenomenon. Through the optimization of BTT, the Li–S battery achieved a specific discharge capacity of up to 1239 mAh g^−1^ during its second cycle. Notably, even after undergoing 300 cycles at a rate of 1C, the battery consistently maintained a high discharge capacity of 907 mAh g^−1^. Furthermore, it exhibited an impressive capacity retention rate and coulombic efficiency of 87.6% and 90%, respectively (Fig. 9f).^116^ In brief, the presence of BTT not only enhances the discharge capacity and cycling stability of Li–S batteries but also endows them with enhanced coulombic efficiency and rate capability. Therefore, the utilization of BTT additives is crucial for addressing interface instability and shuttle effects observed in Li–S batteries, thereby expediting their technological advancement.

In 2020, Wei *et al.* achieved significant improvements in the efficiency of LMBs by introducing an electrolyte additive known as BTB (3,5-bis(trifluoromethyl)thiophenol).^117^ The active thiol groups within BTB interact with lithium metal to form an SEI layer containing organic Ph–S components, providing an effective barrier against LiPSs. This dedicated SEI layer serves the purpose of safeguarding the lithium metal anode against harmful interactions with LiPSs, while simultaneously reducing the utilization of new lithium and electrolyte throughout its operation. As a result, it significantly improves the uniformity of lithium deposition (Fig. 9g) and lowers the overpotential during plating/stripping processes. Under specific testing conditions, including high loading sulfur cathodes (4.5 mg_s_ cm^−2^), low electrolyte-to-sulfur ratios (E/S; 5 μL mg_s_^−1^), and ultra-thin lithium anodes (50 μm), batteries incorporating the organosulfur-inclusive SEI demonstrated up to 82 cycles before experiencing rapid degradation, as compared to only 42 cycles for batteries utilizing conventional SEIs (Fig. 9h). The Li–S batteries with the BTB additive exhibited a high specific discharge capacity of 950 mAh g^−1^ after the initial cycle and retained a capacity of 700 mAh g^−1^ even after 82 cycles, at a current density of 0.1C.^117^ The aforementioned evidence highlights the effectiveness of BTB additives in significantly enhancing both the discharge capacity and cycle stability of Li–S batteries.

**Fig. 10:**
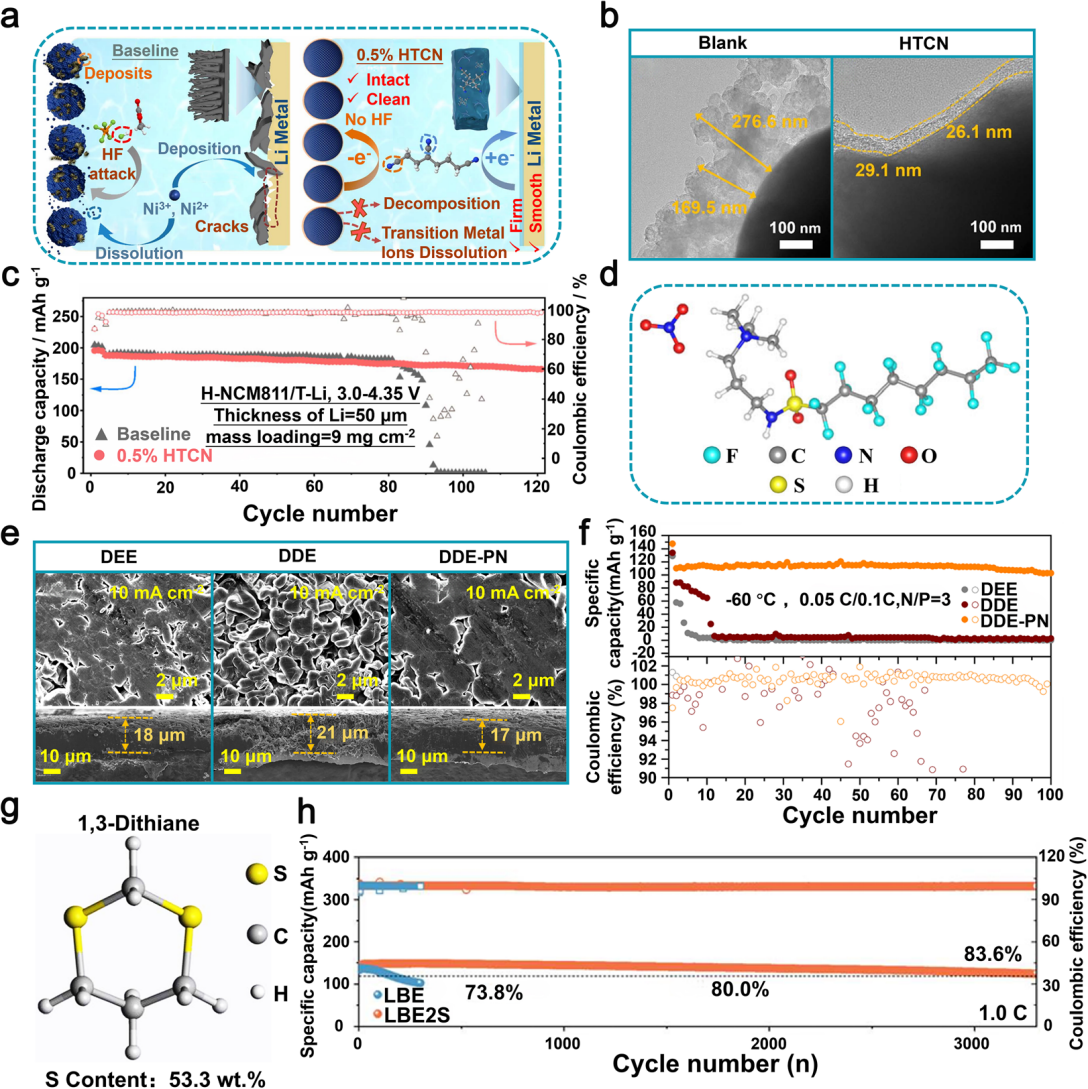
(a) Schematic illustration of the underlying mechanisms of baseline and HTCN-containing electrolytes. (b) TEM images of NCM811 electrodes recovered from L-NCM811‖Li batteries after 100 cycles in baseline electrolyte and HTCN-containing electrolyte. (c) Cycling stability of H-NCM811‖L-Li full batteries in various electrolytes at 0.2C for the initial three cycles and 1C for the subsequent cycles. Reproduced with permission from ref. 118. Copyright 2025, Royal Society of Chemistry. (d) The molecular structural formula of PQA-NO_3_. The cyan spheres represent fluorine (F) atoms, the gray spheres represent carbon (C) atoms, the blue spheres represent nitrogen (N) atoms, the red spheres represent oxygen (O) atoms, the yellow spheres represent sulfur (S) atoms, and the white spheres represent hydrogen (H) atoms. (e) The SEM morphologies and cross-section SEM images of Li deposited on Cu foil cycled in DEE, DDE, and DDE-PN electrolytes under 25 °C. After 5 Li deposition/stripping cycles, a fixed capacity of 2.0 mAh cm^−2^ was deposited on the Cu at a current density of 10.0 mA cm^−2^. (f) Cycling performance of full batteries at −60 °C. Reproduced with permission from ref. 119. Copyright 2025, Springer Nature. (g) Structural formula of 1,3-dithiane. (h) Galvanostatic cycling at 1.0C in Li‖LFP full batteries. Reproduced with permission from ref. 120. Copyright 2025, Oxford University Press.

In 2025, Yang *et al.* reported a novel multi-functional nitrile-based electrolyte additive, 1,3,6-hexanetricarbonitrile (HTCN), designed to enhance the stability and performance of LMBs using high-voltage nickel-rich NCM811 cathodes.^118^ HTCN possesses both linear and side-chain nitrile groups, exhibiting strong affinity towards transition metal ions on the cathode surface, allowing preferential adsorption and oxidation to form a stable CEI (Fig. 10a). Simultaneously, HTCN has lower electron affinity, which facilitates its preferential reduction at the lithium anode, forming a robust SEI (Fig. 10b). This additive effectively stabilizes ion transport kinetics at both electrodes, significantly suppressing dendrite formation and electrolyte decomposition.^150,151^ Electrochemical testing demonstrated remarkable improvements in cycling stability and rate capability. Specifically, batteries employing HTCN-containing electrolytes exhibited a significantly enhanced capacity retention of 88% after 120 cycles at 1C, achieving a high energy density of approximately 330 Wh kg^−1^ at the battery level (Fig. 10c). These findings underscore HTCN's potential as a powerful multifunctional additive for advanced high-energy lithium metal battery applications.

In 2025, Zhang *et al.* developed a multifunctional electrolyte additive, perfluoroalkylsulfonyl quaternary ammonium nitrate (PQA-NO_3_), specifically designed to enhance the performance of LMBs under ultra-low-temperature conditions.^119^ The unique molecular structure of PQA-NO_3_, comprising both cationic (PQA^+^) and anionic (NO_3_^−^) components (Fig. 10d), significantly enhances electrolyte properties by addressing key challenges including sluggish ion transport and lithium dendrite formation. The cationic PQA^+^ component preferentially undergoes *in situ* reduction on lithium metal, forming an inorganic-rich SEI layer characterized by high concentrations of LiF, Li_3_N, Li_2_O, and Li_2_S. This SEI layer demonstrates superior mechanical stability and rapid lithium-ion transport, effectively suppressing dendrite growth (Fig. 10e). Meanwhile, the NO_3_^−^ component modifies the electrolyte's solvation structure, minimizing Li^+^–solvent interactions and significantly reducing the de-solvation energy barrier.^152^ This optimized solvation structure enhances electrolyte ionic conductivity and stability at high voltages, enabling stable operation with high-voltage cathodes such as NMC811. Electrochemical evaluations show exceptional low-temperature performance, with Li‖NMC811 coin batteries demonstrating stable cycling at −60 °C (Fig. 10f). Furthermore, a pouch battery configuration achieved a remarkable specific energy of 171.8 Wh kg^−1^ at −85 °C, retaining 48.1% of its room temperature capacity. Notably, the pouch batteries also exhibited high-rate capabilities, delivering a specific power of 938.5 W kg^−1^ at a discharge rate of 3.0C and −50 °C. These results underscore the substantial potential of PQA-NO_3_ as an advanced additive for high-power, ultra-low-temperature lithium metal battery applications.

In 2025, Xiong *et al.* reported a cyclic thioether additive, 1,3-dithiane, as a promising electrolyte additive to significantly enhance the stability and performance of LMBs.^120^ Due to its unique molecular structure with a high sulfur content (53.5 wt%) (Fig. 10g), 1,3-dithiane preferentially decomposes on both the anode and cathode interfaces, leading to the formation of robust, inorganic-rich interphases. At the anode, 1,3-dithiane facilitates the formation of an SEI layer rich in inorganic components, particularly LiF and Li_2_S. These inorganic-rich interfaces significantly boost lithium-ion transport kinetics, effectively suppress dendrite formation, and reduce active lithium loss during cycling.^153,154^ Simultaneously, the strong interaction between 1,3-dithiane and PF_6_^−^ anions promotes PF_6_^−^ decomposition, contributing to a stable and highly conductive LiF-rich interface. Moreover, the reactive hydrogen atom in 1,3-dithiane interacts with alkyl lithium species to form a sulfur-rich SEI, effectively converting unstable organic components into beneficial inorganic sulfides, thereby improving resistance to solvent decomposition and enhancing SEI stability. In practical Li‖LiFePO_4_ full batteries employing an electrolyte containing 2.0 wt% 1,3-dithiane, outstanding cycling stability was demonstrated, retaining 83.6% of the initial capacity retained after 3300 cycles at 1.0C (Fig. 10h). Furthermore, Ah-level pouch batteries incorporating the 1,3-dithiane additive exhibited exceptional cycling stability with a capacity retention of 93.1% after 150 cycles, demonstrating significant potential for practical applications.

The application of HFPTF effectively enhances the refinement of the primary solvation structure of Li-ions in the electrolyte, resulting in the formation of a more condensed and stable SEI layer, thereby significantly enhancing the performance of LMBs. Similarly, the integration of BTT and BTB effectively addresses the challenges associated with interface instability at the lithium-metal anode and the migration of soluble LiPSs in Li–S batteries. Recent studies have further demonstrated that HTCN significantly stabilizes interfaces by forming stable inorganic-rich SEI layers and CEIs, effectively suppressing dendrite growth and enhancing battery cycle life. Similarly, PQA-NO_3_ uniquely addresses ultra-low-temperature performance challenges by forming a robust inorganic-rich SEI and optimizing the electrolyte solvation structure, greatly improving battery stability under extreme conditions. The additive 1,3-dithiane, characterized by its high sulfur content, also contributes to the formation of a durable and conductive SEI, significantly reducing dendrite formation and active lithium loss. Beyond the well-studied ester, organic sulfides, and carboxylic acid additives, sulfonic acid-, alcohol-, and phenol-based additives also make substantial contributions to stabilizing the SEI layer, providing robust protection to the lithium-metal anode. Overall, the utilization of small organic molecules as additives in LMBs has multiple advantages. Firstly, owing to their low molecular weight and high reactivity, these organic molecules can rapidly undergo reactions with the lithium-metal surface, resulting in the formation of a homogeneous and stable SEI layer. This SEI layer effectively isolates the lithium-metal from undesired side reactions with the electrolyte, thereby prolonging the battery's lifespan. Secondly, these organic molecules exhibit excellent solubility and conductivity in the electrolyte, thereby enhancing the overall electrolyte conductivity and ion transport efficiency. Finally, these substances enhance the SEI layer's durability and resistance to heat, reducing the likelihood of electrolyte breakdown and enhancing the battery's safety characteristics.

## 4. Major molecular additives

Organic high molecular weight additives, typically with a molecular weight exceeding 1000, exhibit superior chemical and thermal stability compared to low molecular weight counterparts. The macromolecular additives facilitate the formation of a robust SEI layer, thereby enhancing the thermal resilience of LMBs and effectively suppressing the growth of lithium dendrites. Additionally, their abundant functional groups can form stable hydrogen or ionic bonds with Li-ions, thereby enhancing both the conductivity and overall stability of the battery system (Table 2). Our research focuses on applying these high molecular weight additives in LMBs, primarily emphasizing their roles in safeguarding the lithium metal anode and boosting battery performance based on insights from proteins and polymers.

**Table 2:** The application of macromolecular electrolyte additives in rechargeable batteries

Organic additives	Applications	Cathodes	Anodes	Electrode	Electrolyte	Electrochemical performances	Ref.
Zein	Lithium metal battery	LiFePO_4_	Li	80 wt% LiFePO_4_ + 10 wt% super C45 and 10 wt% PVDF in *N*-methy-l-2-pyrrolidone (NMP)	1 M LiPF_6_ in EC/DMC + 0.05 wt% zein	130.1 mAh g^−1^ at 1C after 200 cycles	155
Hemoglobin	Lithium–air battery	Nickel foam	Li	90 wt% MWCNT + 10 wt% PVDF on the nickel foam	Hemoglobin in 1 M LiClO_4_ + TEGDME	500 mAh g_CNT_^−1^ between 4.5 and 1.75 V at 100 mA g_CNT_^−1^	156
Fibroin	Lithium metal battery	Li_4_Ti_5_O_12_	Li	80 wt% BTR Co + 10 wt% CB + 10 wt% CMC in the LTO electrode	1 M TFSI in DOL/DME + 1 wt% LiNO_3_ + 0.5 wt% fibroin	125 mAh g^−1^ at 350 mA g^−1^ after 2000 cycles	157
PAN	Lithium metal battery	LiFePO_4_	Li	70 wt% LiFePO_4_ + 15 wt% PVDF + 15 wt% Super-P in NMP solvent	1 M LiTFSI in DMF + PAN/PEO (1 : 4) + 5 wt% LAO	167 mAh g^−1^ at 0.1C after 25 cycles	158
Zn(BEH)_2_	Lithium metal battery	LiFePO_4_	Li	80 wt% LiFePO_4_ + 10 wt% Super-P + 10 wt% PEO/LiTFSI in NMP	LiTFSI + Zn(BEH)_2_ + PEO in AN (EO : Li^+^ : Zn^2+^ = 20 : 1 : 1)	135 mAh g^−1^ at 0.2C after 100 cycles	159
OP-10	Lithium metal battery	LiFePO_4_	Li	70 wt% LiFePO_4_ + 20 wt% carbon black + 10 wt% PVDF in NMP	1 M LiPF_6_ in PC/EC/DEC (1 : 4 : 5) + 5 wt% OP-10	67.1 mAh g^−1^ at 10C after 1000 cycles	160
HPMC	Lithium metal battery	LiFePO_4_	Li	80 wt% LiFePO_4_ + 10 wt% acetylene black + 10 wt% LA-132 binder	1 M LiPF_6_ in EC/DMC/EMC (1 : 1 : 1) + 50 wt% HPMC	153 mAh g^−1^ at 0.2C after 50 cycles	161
PST	Lithium metal battery	S	Li	KB with 70 wt% S	1 M LiTFSI + 4 wt% LiNO_3_ in DOL/DME (1 : 1) + 8 wt% PST-90 (90 wt% S)	735 mAh g^−1^ at 1672 mA g^−1^ after 1000 cycles	162
HFPP	Lithium metal battery	CNT/S	Li	80 wt% CNT/S (66 wt%) + 15 wt% SP + 5 wt% LA133	1 M LiTFSI + 1% LiNO_3_ in DOL/DME (1 : 1) + 5% HFPP	763 mAh g^−1^ at 837.5 mA g^−1^ after 200 cycles	163
PSD	Lithium metal battery	PSD	Li	PSD	P(VDF-HFP) + 10% PSD	780 mAh g^−1^ at 167.5 mA g^−1^ after 200 cycles	164
NiDME	Lithium metal battery	CMK-3/S	Li	80 wt% CMK-3/S (70% S) + 10 wt% Super P + 10 wt% PVDF	1 M LiTFSI + 1% LiNO_3_ in DOL/DME (1 : 1) + 0.5 wt% NiDME	784 mAh g^−1^ at 1C after 500 cycles	165
PVDF	Lithium–air battery	Graphene/α-MnO_2_	Li	Graphene + 30 wt% α-MnO_2_	1 M LiPF_6_ in TEGDME + PEO + PVDF	4488 mAh g^−1^ at 1st cycles	166
SCS	Lithium metal battery	NCM523	Li	80 wt% NCM523 powder + 10 wt% acetylene black + 10 wt% PVDF in NMP	0.4517 g PVDF + 0.0226 g SCS + 0.301 g LiFSI in 7 ml THF and 3 ml DMA	130.5 mAh g^−1^ at 0.2C after 50 cycles	167

### 4.1. Protein additives

To date, numerous organic additives, such as fluorinated 2-fluoropyridine (2-FP) and sulfur-containing dimethyl sulfate (DMS), have failed to meet expectations in their application within LMBs.^168–170^ While these additives may alter the composition of the SEI and the solvation sheath,^171^ they also risk generating toxic hydrogen fluoride (HF) gas,^172–174^ thereby increasing safety concerns regarding the batteries. Therefore, it is of utmost significance to focus on the creation of electrolyte additives that are both environmentally friendly and economically viable in order to facilitate the widespread implementation of LMBs. In the past few years, there has been a growing trend in research utilizing non-toxic proteins as electrolyte additives.^175,176^ These proteins are not only cost-effective and easy to process but also minimally disruptive to the internal battery environment.^177,178^ Most notably, as representative bio-macromolecules, proteins inherently possess a wealth of functional groups at the molecular level, showcasing tremendous potential as electrolyte additives in enhancing battery performance.^179,180^ Protein additives are predominantly explored in aqueous or GPEs due to their excellent solubility, biocompatibility, and rich functional group chemistry, which enables strong interactions with lithium ions and effective modulation of the electrode/electrolyte interface.

**Fig. 11:**
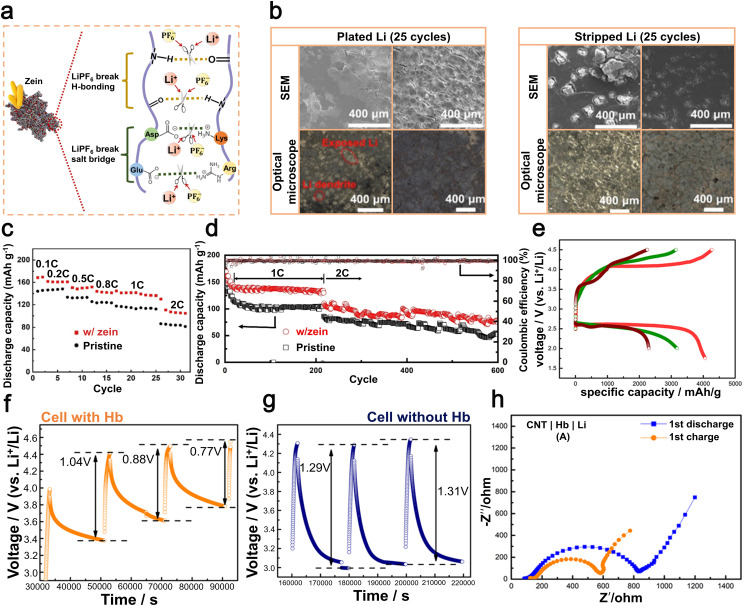
(a) Schematic diagram illustrating the denaturation process of zein, a class of prolamine protein found in maize. (b) Surface morphology of Li‖Li cells with and without zein, coated with Li, as observed using SEM and optical microscopy; surface morphology of Li deposition on Li‖Li cells with and without zein as observed using SEM and optical microscopy. (c) Rate performance of Li‖LiFePO_4_ full cells at different current densities, with and without the addition of zein. (d) The extended cycling durability of Li‖LiFePO_4_ full cells with and without zein. Reproduced with permission from ref. 155. Copyright 2022, Elsevier. (e) Lithium–air batteries were subjected to charge–discharge cycles with the addition of redox media hemoglobin (red) and hemoglobin (green), as well as without any redox medium, using bare carbon nanotubes (brown). The current was kept constant throughout the experiment. Galvanostatic Intermittent Titration Technique (GITT) applied to lithium–air batteries assembled (f) with hemoglobin as the redox medium (orange) and (g) without a redox medium (blue). (h) The electrochemical impedance spectrum of lithium–air batteries is presented for the first charge (blue) and discharge (orange). Reproduced with permission from ref. 156. Copyright 2019, American Chemical Society.

Proteins consist of numerous amino acids and possess polar functional groups, including –CO–NH–, –COOH, –OH, and –NH_2_, which are embedded within their structure. These functional groups are capable of participating in diverse interactions with the electrolyte solvents and lithium salts,^181^ which have inspired applications of proteins in adhesives,^182,183^ membrane coatings,^184,185^ and protective layers^186–188^ for lithium anodes. In 2022, Wang *et al.* exploited zein proteins from abundant natural corn as a novel additive.^155^ Natural proteins are macromolecules with a quaternary structure that conceals a multitude of functional groups. Wang *et al.* denatured zein proteins into their secondary or primary structures in an EC/EMC/LiPF_6_ electrolyte solution. In EC/EMC, a high concentration of LiPF_6_ (1 M) disrupts the salt bridges and hydrogen bonds in zein. This leads to exposure to various polar functional groups (Fig. 11a).^114,189^ The LUMO and HOMO levels of EC, EMC, and protein molecules were determined by Wang *et al.* through first-principles calculations. They discovered that peptides with lower LUMO levels preferentially undergo reduction at the Li anode surface before EC and EMC, thereby participating in the formation of the SEI and reducing the consumption of electrolyte solvents. The SEM and optical microscopy analysis revealed that the zein protein-enriched SEI facilitates the immobilization of anions, thereby enabling uniform deposition of Li-ions and suppression of dendrite formation after 25 cycles (Fig. 11b). The preferential reaction of zein proteins with lithium effectively repairs the cracked SEI and prevents the lithium anode from being corroded by liquid carbonate electrolytes (Fig. 11b). Moreover, the inclusion of zein proteins alters the functional groups in the lithium anode's SEI, resulting in reduced polarization during cycling. As a result, the cycling lifespan of symmetric lithium batteries is significantly increased from less than 200 hours to over 350 hours when operating at a capacity of 1 mAh cm^−2^ and a current density of 1 mA cm^−2^. When combined with a LiFePO_4_ cathode, the incorporation of the zein protein additive leads to notable enhancements in electrochemical performance, encompassing rate capability and long-term cycling stability. Li‖LiFePO_4_ full batteries exhibit a discharge capacity of around 168.3 mAh g^−1^ at a rate of 0.1C (Fig. 11c). Following 200 cycles at a rate of 1C, the capacity remains at 130.1 mAh g^−1^, which corresponds to an impressive retention rate of approximately 80% (Fig. 11d).^155^ This study suggests that zein, a type of natural protein, can be utilized as a highly efficient electrolyte additive in carbonate-based LMBs to significantly improve their performance and lifespan.

Hemoglobin, a protein consisting of four subunits, is found in the blood and plays a crucial role in transporting oxygen molecules. The active site of this protein contains iron-porphyrin and is surrounded by α-helical amino acid chains, which form a secondary structure. Additionally, the tertiary structure of hemoglobin comprises four similar subunits.^190^ In 2019, Samajdar *et al.* and colleagues employed hemoglobin as a bio-oxygen adhesive/transport protein to enhance the performance of non-aqueous lithium–air batteries.^156^ Reproducing the process through which hemoglobin attaches to and carries oxygen in human blood, their aim was to utilize this mechanism in the electrolyte of conventional lithium–air batteries to facilitate oxygen attachment and transportation. The research discovered that enhancing the performance of the oxygen reduction reaction (ORR) at the air cathode involved inhibiting the buildup of solid insulating discharge byproducts near the cathode site and facilitating oxygen transfer to the soluble electrolyte phase. In the presence of hemoglobin, N. Samajdar *et al.* observed stable constant-current cycling (Fig. 11e) and low polarization (Fig. 11f and g). Electrochemical impedance spectroscopy indicated (Fig. 11h) that the interfacial resistance remained low even after several constant-current charge–discharge cycles.^156^ Therefore, it is highly anticipated and feasible to use natural protein molecules as redox mediators in the electrolyte for oxygen electrochemistry in future energy harvesting systems.

**Fig. 12:**
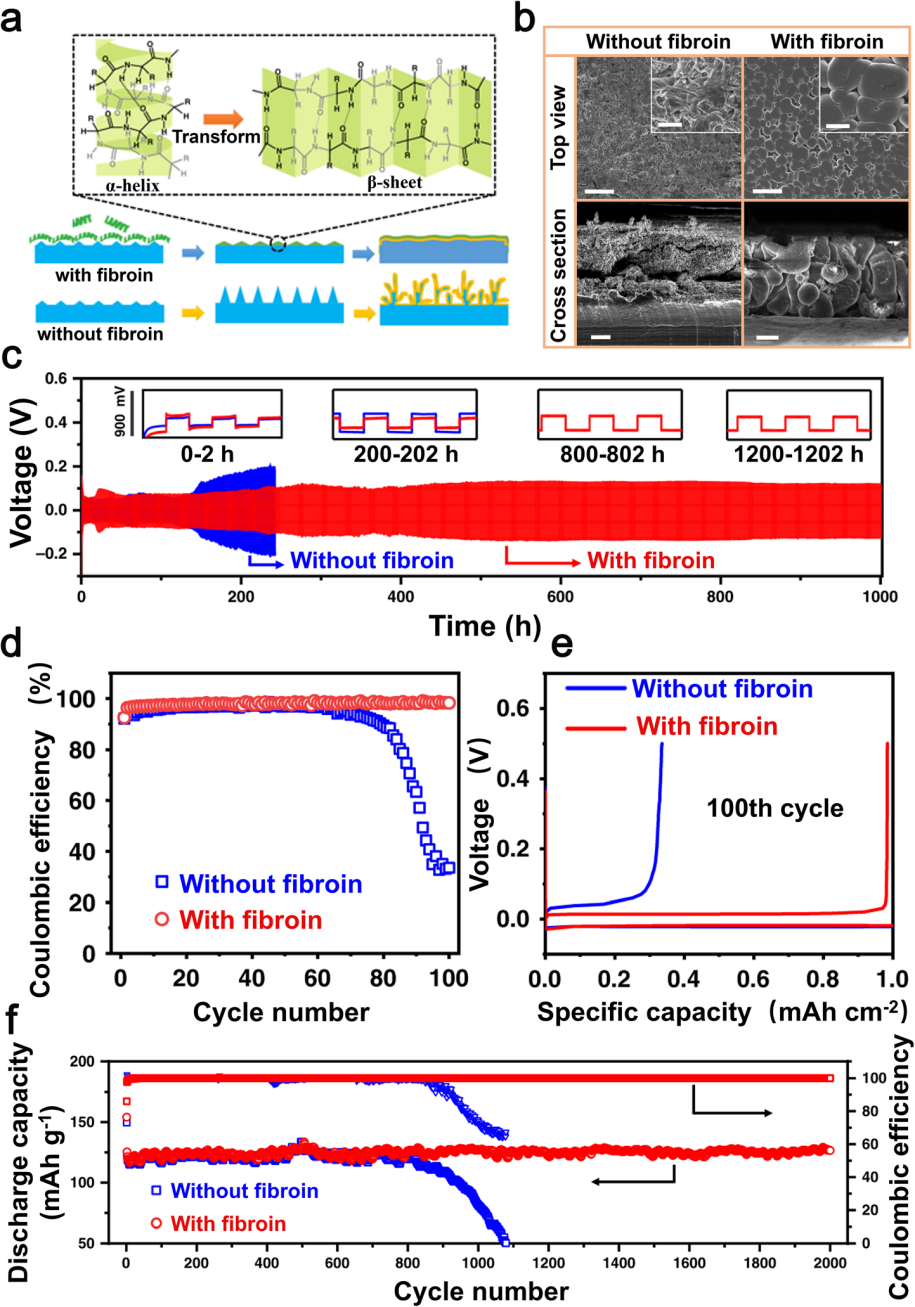
(a) The alteration of the secondary configuration of silk protein and the self-protective mechanism of immune lithium metal anode against the growth of lithium dendrites. (The “R” groups are referred to as side chains of amino acids, where –H represents the glycine residue, –CH_2_OH corresponds to the serine residue, and –CH_3_ signifies the alanine residue). (b) Top-view and cross-sectional SEM images of the cycling lithium metal anode in batteries with blank electrolyte and electrolyte containing silk protein. (c) Constant current cycling of symmetric Li‖Li batteries with or without the inclusion of silk protein. (d) Comparison of cycling performances of Li‖Cu half-cells with or without a fibroin interlayer between Cu foil and the separator. The amount of Li deposited in each cycle is 1 mAh cm^−2^ and the current density is 1 mA cm^−2^. (e) The corresponding voltage profiles at the 100th cycle of the Li plating/stripping processes on Cu foil with or without a fibroin interlayer. (f) Li‖LTO full batteries were subjected to long-term cycling stability tests at a current density of 2C (1C = 175 mA g^−1^), with and without the incorporation of silk protein, in order to minimize the likelihood of plagiarism. Reproduced with permission from ref. 157. Copyright 2020, Springer Nature.

Currently, there exist two categories of electrolyte additives utilized in lithium metal anodes: the initial category actively engages in the creation of the SEI, thereby greatly augmenting its physical characteristics and chemical durability.^89^ The second category attaches to the ends of lithium protrusions, creating a shield with a positive electrostatic charge around them. This effectively directs additional lithium deposition towards neighboring regions, thereby reducing the occurrence of dendrite formation.^191,192^ The previously mentioned corn protein and hemoglobin belong to the first category; next, we discuss the second. In 2020, Wang *et al.* discovered that the incorporation of natural silk fibroin as an electrolyte additive exhibits remarkable inhibitory effects on dendrite growth and effectively eliminates minuscule dendritic particles.^157^ As a result, this protein-based approach significantly enhances the longevity of the lithium metal anode's cycling performance while concurrently improving its coulombic efficiency. The surface of the lithium metal anode, especially the tips of lithium buds, is naturally coated with silk fibroin molecules. This occurs due to their spatial conformation and transformation from an α-helix to a β-sheet structure (Fig. 12a), facilitating adsorption.^193,194^ This effectively modifies the distribution of the electric field surrounding the tips of lithium buds, resulting in even electrodeposition and stripping while inhibiting the growth of mossy lithium into slender dendrites.

Through SEM, Wang *et al.* observed that lithium anodes with added silk fibroin displayed a dense, uniform, nodular morphology of Li deposition after 15 cycles (Fig. 12b), contrasting sharply with dendritic growth in anodes without silk fibroin. This visual observation provides evidence that the inclusion of silk fibroin in the electrolyte successfully inhibits the growth of mossy lithium during its early stages, thereby preventing the formation of slender dendrites. Silk fibroin-incorporated lithium symmetric batteries demonstrated a consistent voltage profile throughout 1000 hours of operation, maintaining a stable cycle during plating and stripping processes (Fig. 12c), while operating at a current density of 3 mA cm^−2^ and achieving a capacity of 1 mAh cm^−2^. The coulombic efficiency of Li‖Cu half batteries was also investigated by Wang *et al.* in order to evaluate the impact of the additive on cycle life. The results showed that Li‖Cu half batteries containing silk fibroin maintained a remarkable coulombic efficiency of up to 98% even after undergoing 100 cycles at a current density of 1 mA cm^−2^ (Fig. 12d and e). Additional analysis was conducted on Li‖Li_4_Ti_5_O_12_ (LTO) full batteries, revealing that the inclusion of silk fibroin resulted in enhanced cycling stability and rate performance. The silk fibroin-infused batteries exhibited excellent capacity retention and maintained nearly perfect coulombic efficiency throughout 2000 cycles. At a current density of 2C, these batteries demonstrated an impressive specific capacity of approximately 125 mAh g^−1^ after undergoing 2000 cycles, surpassing the performance of Li‖LTO full batteries with unmodified electrolyte (Fig. 12f).^157^

### 4.2. Polymer additives

Polymer additives exhibit remarkable performance particularly in solid-state and GPEs due to their compatibility with polymer matrices and the capacity to form robust, mechanically stable interfacial layers. Using high-molecular-weight polymers as additives can significantly enhance the structural stability of the SEI. This enhanced stability can be primarily attributed to the polymer chains that strengthen the SEI layer, thereby enhancing its resistance against lithium dendrite infiltration. Moreover, the highly cross-linked polymers exhibit excellent thermal and mechanical stability, which further contributes to maintaining the integrity of the SEI layer. These polymer additives have the ability to form a protective layer on top of the lithium metal, which can effectively hinder or impede the formation and expansion of lithium dendrites. Consequently, the utilization of polymers as electrolyte additives holds tremendous potential in the development of LMBs.

**Fig. 13:**
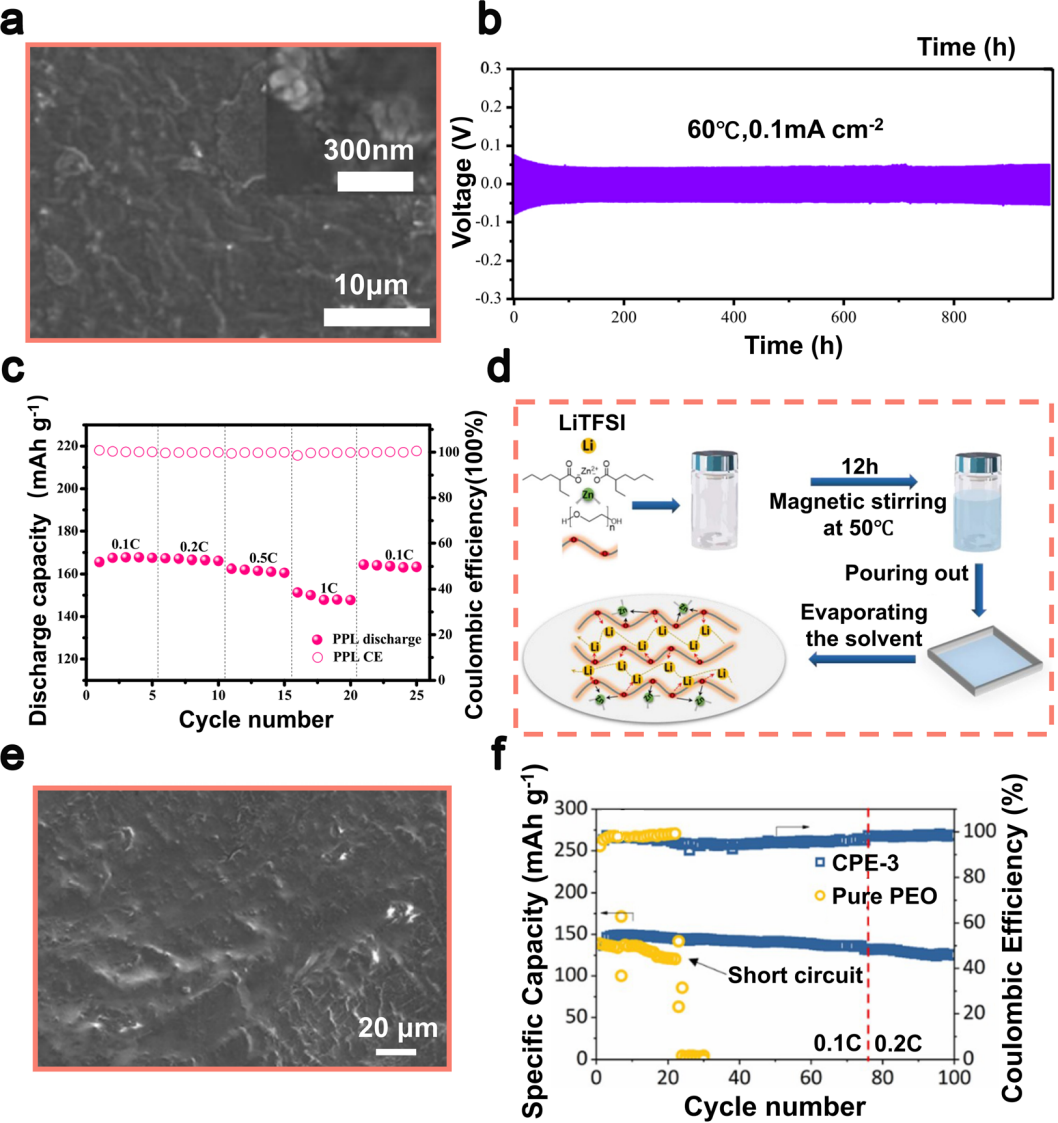
(a) SEM image of the P-P-L surface (inset: P-P-L image under a high magnification scanning microscope). (b) Voltage distribution graph of the P-P-L electrolyte symmetric battery at 0.1 mA cm^−2^ current density at 60 °C. (c) Rate performance of the LiFePO_4_|P-P-L|Li full battery. Reproduced with permission from ref. 158. Copyright 2022, Elsevier. (d) Schematic diagram of the preparation process of PEO-Zn(BEH)_2_. (e) SEM images of the lithium foil surface cycled in CPE-3 SPEs. (f) Cycling performance of LiFePO_4_|SPE|Li and LiFePO_4_|CPE-3|Li at 0.1C and 0.2C. Reproduced with permission from ref. 159. Copyright 2020, Elsevier.

Solid electrolytes, characterized by their low flammability, excellent thermal stability, and mechanical robustness, have garnered considerable attention from researchers.^195–197^ These electrolytes are primarily categorized into three types: inorganic ceramic electrolytes (ICEs),^198,199^ SPEs,^200^ and CSEs.^201^ Currently, the research is primarily focused on CSEs, which combine the benefits of inorganic and polymer electrolytes. However, their performance still requires further enhancement to meet practical applications.^202,203^ In 2022, Ning Lv and his team combined the advantages of both inorganic and organic additives by incorporating LiAlO_2_ (LAO) and polyacrylonitrile (PAN) into a polyethylene oxide (PEO)-based composite solid electrolyte (P-P-L).^158^ PAN is notable for its high electrochemical stability and its unique –C≡N groups,^204^ which facilitate the effective transport of Li-ions and mitigate lithium dendrite formation (Fig. 13a).^166,168^ Additionally, this material can form Li_3_N on the surface of lithium metal, significantly reducing interfacial impedance and consequently boosting battery performance.^205^ After performing charge–discharge experiments at different current densities, it was observed that the Li|SE|Li battery exhibited stable operation for approximately 1000 hours at a current density of 0.1 mA cm^−2^ and a temperature of 60 °C (Fig. 13b). Additionally, at 0.1C current density, the battery exhibited an outstanding specific discharge capacity of 167 mAh g^−1^ (Fig. 13c).^158^ These findings unequivocally suggest that the P-P-L composite electrolyte with added PAN has considerable potential for future applications in LMBs.

While all-solid-state LMBs have utilized composite solid electrolytes to some extent, SPEs incorporating PEO are also regarded as highly suitable options for the construction of such batteries.^206,207^ However, their widespread adoption has been impeded due to limitations such as insufficient ionic conductivity, inadequate mechanical strength, and the inability to effectively suppress the formation of lithium dendrites.^208,209^ To overcome these challenges, a study by Zeng *et al.* in 2020 employed a simple solution casting technique to introduce zinc bis(2-ethylhexanoate) (Zn(BEH)_2_) as an additive into the PEO matrix (Fig. 13d), followed by extensive physicochemical and electrochemical characterization.^159^ The interaction between Zn(BEH)_2_ and PEO, known as Lewis acid–base interaction, effectively reduced the crystallinity of the polymer. As a result, the ionic conductivity was significantly improved at both 30 °C and 60 °C, with measured values of 1.1 × 10^−5^ S cm^−1^ and 2.7 × 10^−4^ S cm^−1^, respectively. This advancement also increased the lithium-ion transference number to 0.5. Batteries constructed with the modified PEO-Zn(BEH)_2_ electrolyte demonstrated 500 hours of stable cycling and a low polarization voltage of 150 mV. After cycling, the surface of the lithium-metal anode appeared smooth, primarily due to the *in situ* formation of a LiZn alloy layer through the reaction between Zn(BEH)_2_ and lithium. This reaction effectively suppressed the growth of lithium dendrites (Fig. 13e). Furthermore, a full battery consisting of LiFePO_4_ as the cathode, CPE-3 (PEO with Zn(BEH)_2_ additive) as the electrolyte, and lithium metal as the anode was also successfully assembled. This complete battery exhibited an initial cycle-specific capacity of 145 mAh g^−1^ at a current density of 0.1C. Furthermore, it retained a significantly high specific capacity of approximately 135 mAh g^−1^ with an impressive coulombic efficiency of around 99% after undergoing 100 cycles at a current density of 0.2C (Fig. 13f).^159^

**Fig. 14:**
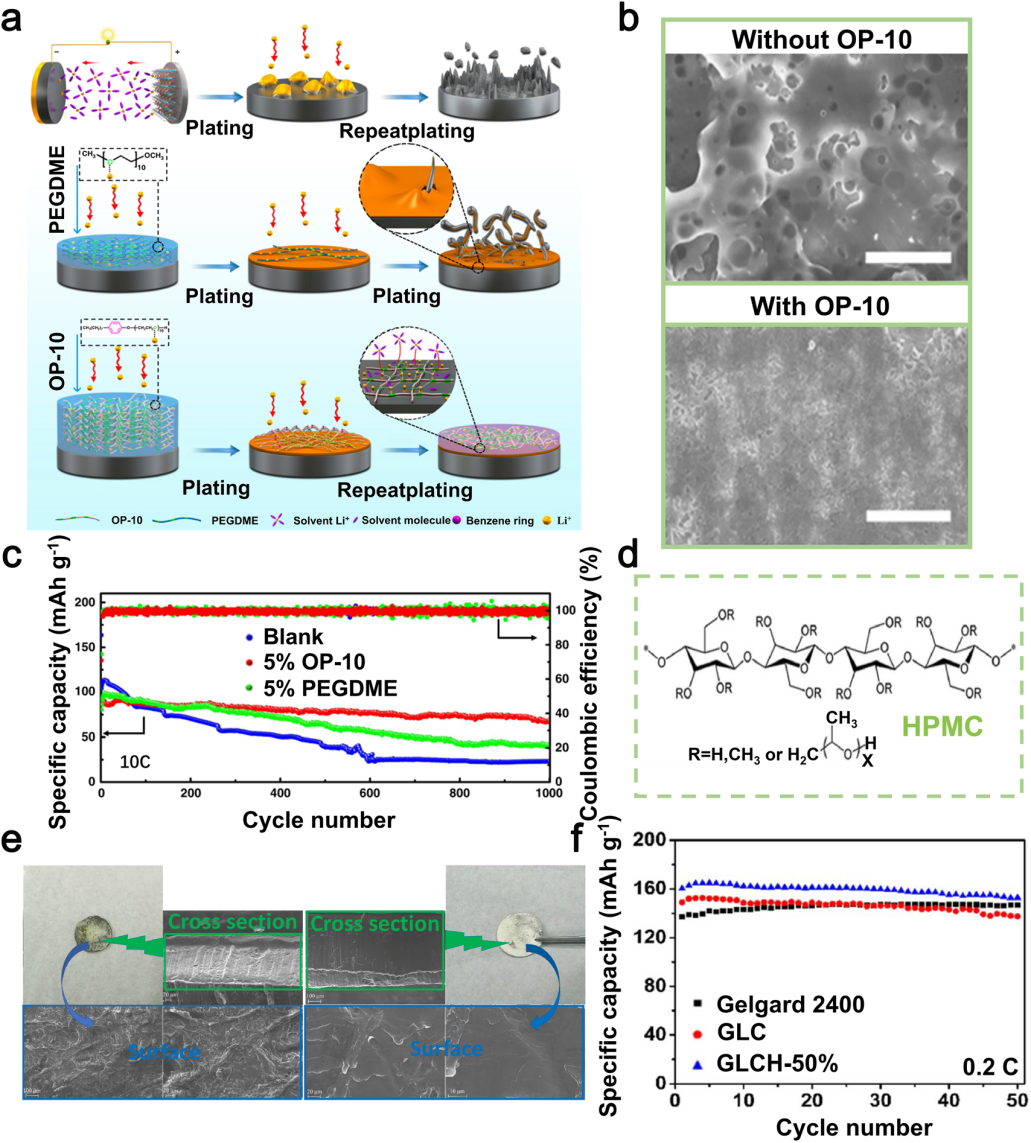
(a) Schematic illustration of the protective mechanism of the additive and lithium deposition with/without various additives. (b) Top-view SEM images of lithium deposition on Cu foil with/without OP-10 at 0.5 mA cm^−2^ for 0.4 h. (c) Long-term cycling performance of Li‖LiFePO_4_ full batteries in blank or modified electrolyte at 10C. Reproduced with permission from ref. 160. Copyright 2020, Springer Nature. (d) Structural diagram of HPMC. (e) Digital photos and SEM images of the lithium electrode after 1000 hours of cycling with/without the HPMC additive. (f) Cycling performance of Li‖LiFePO_4_ full batteries at 0.2C. Reproduced with permission from ref. 161. Copyright 2020, Elsevier.

In 2020, Dai *et al.* effectively stabilized the lithium metal anode by incorporating octaphenyl polyethylene (OP-10) as an electrolyte additive.^160^ The structure of OP-10 contains epoxy ethane (EO) units that display a high affinity towards Li-ions (Fig. 14a). These entities combine with Li-ions, promoting a uniform deposition of lithium and inhibiting the proliferation of lithium dendrites (Fig. 14b). The extensive phenyl chains present in OP-10 establish a network that is interconnected in three dimensions, thereby augmenting the chemical and structural stability of the EO units. Consequently, this prolongs the lifespan of the additive throughout electrochemical cycling. Consequently, a robust and flexible SEI layer forms. This SEI layer serves as a protective shield, effectively minimizing direct interaction between the lithium metal anode and the electrolyte solvent, thereby alleviating solvent degradation and inhibiting dendrite growth. It facilitates high current density lithium stripping and plating operations with long-term stability. The utilization of OP-10 as an additive resulted in remarkable cyclic stability of Li‖Li batteries, with over 400 cycles achieved at a current density of 1 mA cm^−2^. When combined with a LiFePO_4_ cathode in a complete battery setup, the battery exhibited an impressively minimal decline in capacity per cycle, measuring only 0.023%. Furthermore, it maintained a high capacity of 67.1 mAh g^−1^ even after undergoing up to 1000 cycles (Fig. 14c). In summary, the OP-10 compound functions as a highly effective electrolyte additive, facilitating both the homogeneous deposition of lithium and the formation of a stable SEI layer. This impedes the expansion of lithium dendrites to a significant extent, which opens up possibilities for creating lithium-powered batteries that possess enhanced safety features, greater longevity, and increased voltage capacity.^210,211^

The inherent deficiencies of gel polymer electrolytes (GPEs) have posed a bottleneck to the overall performance of LMBs.^212,213^ Despite the incorporation of high quality cellulose-based GPEs, such as lignocellulose (LC), their inadequate mechanical properties, fluid absorption rate, and ionic conductivity remain significant obstacles for practical applications.^214,215^ To mitigate these limitations, in 2020, Luo *et al.* endeavored to integrate a novel, highly swellable, biodegradable hydroxy propyl methyl cellulose (HPMC) polymer into the LC-based polymer matrix.^161^ HPMC, a naturally derived cellulose polymer, possesses attributes of biodegradability and sustainability (Fig. 14d).^216,217^ The excellent film-forming capabilities and solubility of HPMC in water and organic solvents are attributed to the hydroxyl and methoxy groups in its structure, positioning it as a potentially promising GPE composite substrate. The phase transfer method was employed to prepare LCH membranes, which significantly improved the electrochemical properties of GPEs. These membranes exhibited excellent ionic conductivity (6.14 × 10^−3^ S cm^−1^ at 25 °C), impressive charge–discharge capabilities (162 mAh g^−1^ at 0.2C), and remarkable control over lithium deposition (Fig. 14e). In addition, the Li|GLCH-50%|LiFePO_4_ full battery demonstrated a remarkable initial capacity of 162 mAh g^−1^ at 0.2C, while maintaining a capacity of 153 mAh g^−1^ after undergoing 50 cycles (Fig. 14f).^161^ This clearly indicates the substantial improvement in both ionic conductivity and the lithium-ion transference number achieved through the integration of HPMC.

**Fig. 15:**
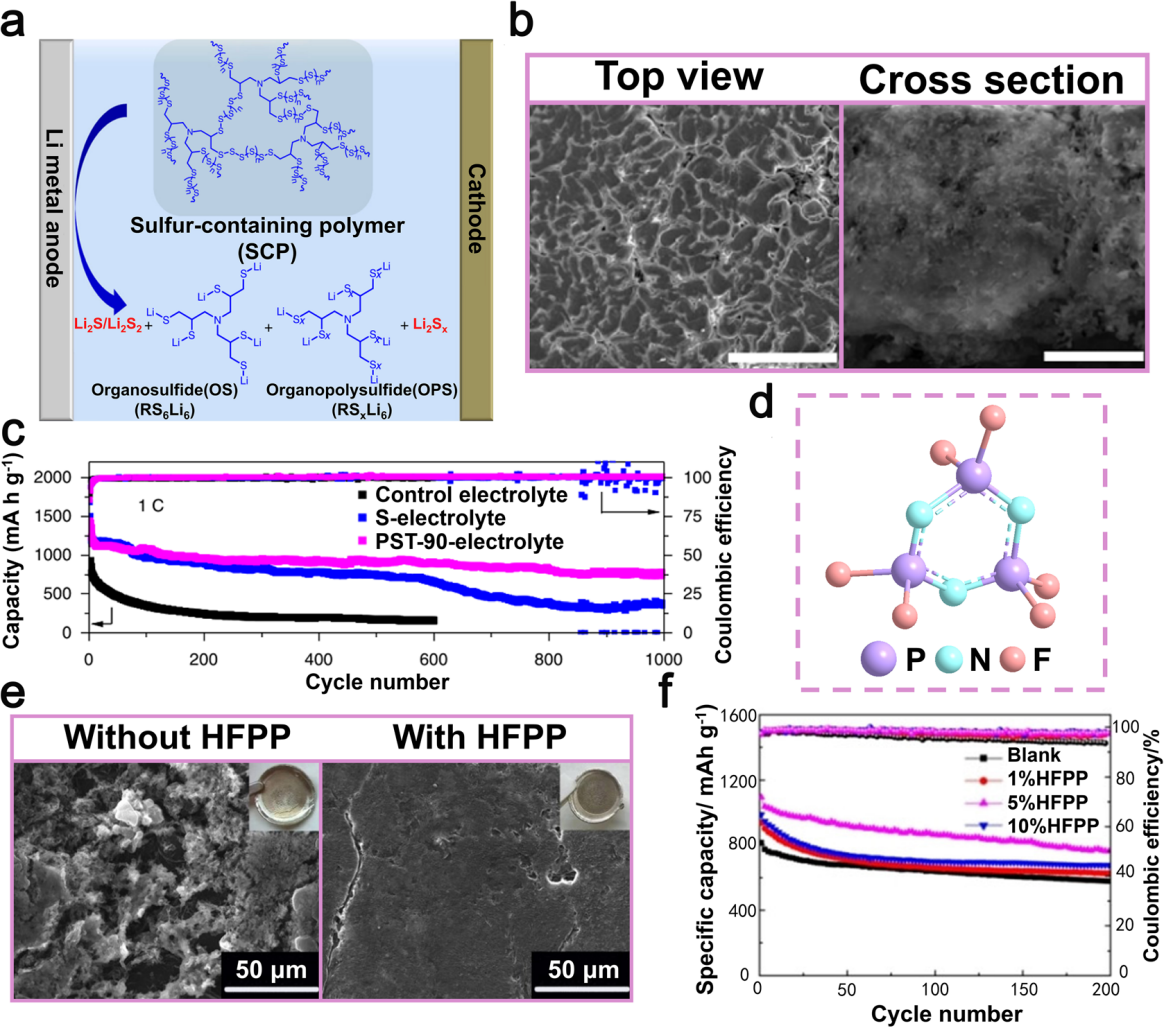
(a) Schematic of the formation of a stable inorganic/organic hybrid SEI layer, SCP provides organic units (organic sulfides/organic polysulfides) and inorganic units (Li_2_S/Li_2_S_2_) in the electrolyte. (b) Morphology of lithium metal deposited on a stainless-steel substrate. (c) Electrochemical performance of Li–S batteries with different additives at a rate of 1C. Reproduced with permission from ref. 162. Copyright 2017, Springer Nature. (d) Chemical structure of HFPP. (e) SEM images of the lithium anode surface after 200 cycles at 0.5C: pristine lithium anode before cycling; electrolyte without HFPP; electrolyte with HFPP. (f) Cycling performance of Li–S batteries with 0 wt%, 1 wt%, 5 wt%, and 10 wt% HFPP in the electrolyte at 0.5C. Reproduced with permission from ref. 163. Copyright 2021, Wiley-VCH.

The impediment to attaining exceptional cycle stability in Li–S batteries lies in the deterioration of lithium metal.^218,219^ The creation of the SEI layer may involve the incorporation of Li polysulfides, which react with lithium metal to produce inorganic elements such as Li_2_S/Li_2_S_2_. This process can result in the dissolution and migration of these compounds.^220,221^ Although such SEI layers somewhat enhance the cycle efficiency,^222,223^ they fail to realize the prolonged cycle life of Li–S batteries. In 2017, Li *et al.* and colleagues dissolved the triallylamine (TAA) monomer directly in liquid sulfur and heat-treated the mixture at 145 °C, co-polymerizing it to produce poly(sulfur-random-triallylamine) (PST).^162^ 90 wt% of PST was used as an additive in the electrolyte, mixed thoroughly, and then injected into the battery, where sulfur-containing polymers (SCP) electrochemically decomposed into Li organic sulfides, Li organic polysulfides, Li_2_S_*x*_, and Li_2_S/Li_2_S_2_ upon coming in contact with lithium metal (Fig. 15a). Organic sulfides and organic polysulfides play a role in increasing the flexibility and stability of the hybrid SEI layer during lithium plating/stripping processes in the inorganic Li_2_S/Li_2_S_2_ phase, resulting in improved CE and reduced formation of lithium dendrites (Fig. 15b). This combination of the SEI layer significantly enhances the battery's coulombic efficiency, resulting in a remarkable coulombic efficiency of 99% even after undergoing over 400 cycles at a current density of 2 mA cm^−2^. At a 1C (1C = 1672 mA g^−1^) discharge rate, the Li–S battery retains a capacity of 735 mAh g^−1^ between the 10–1000 cycles, with a CE of about 99.9% (Fig. 15c).^162^ It is clear that the inclusion of the PST additive enhances the longevity and capacity preservation of Li–S batteries, playing a vital role in ensuring the stable formation of the SEI layer.

In 2021, a hybrid organic–inorganic polymer known as hexafluorocyclo-triphosphazene (HFPP) was integrated into the electrolyte by Chen *et al.*^163^ The polymer demonstrates a planar symmetrical two-dimensional molecular structure (Fig. 15d) and possesses a distinctive electronic configuration, reduced interfacial resistance, and enhanced efficiency in the deposition of Li-ions. The presence of unshared electron pairs on the nitrogen atoms in HFPP promotes consistent diffusion of Li-ions, while its two-dimensional configuration improves the robustness of the SEI, effectively preventing the occurrence of lithium dendrites (Fig. 15e). The Li–S batteries, incorporating HFPP as an electrolyte additive, exhibited a capacity of 1095 mAh g^−1^ at 0.5C (1C = 1675 mA g^−1^), along with a reversible capacity of up to 763 mAh g^−1^ and a consistently high coulombic efficiency of around 98% (Fig. 15f).^163^ This underscores HFPP as an efficacious additive capable of effectively protecting the lithium anode SEI layer.

**Fig. 16:**
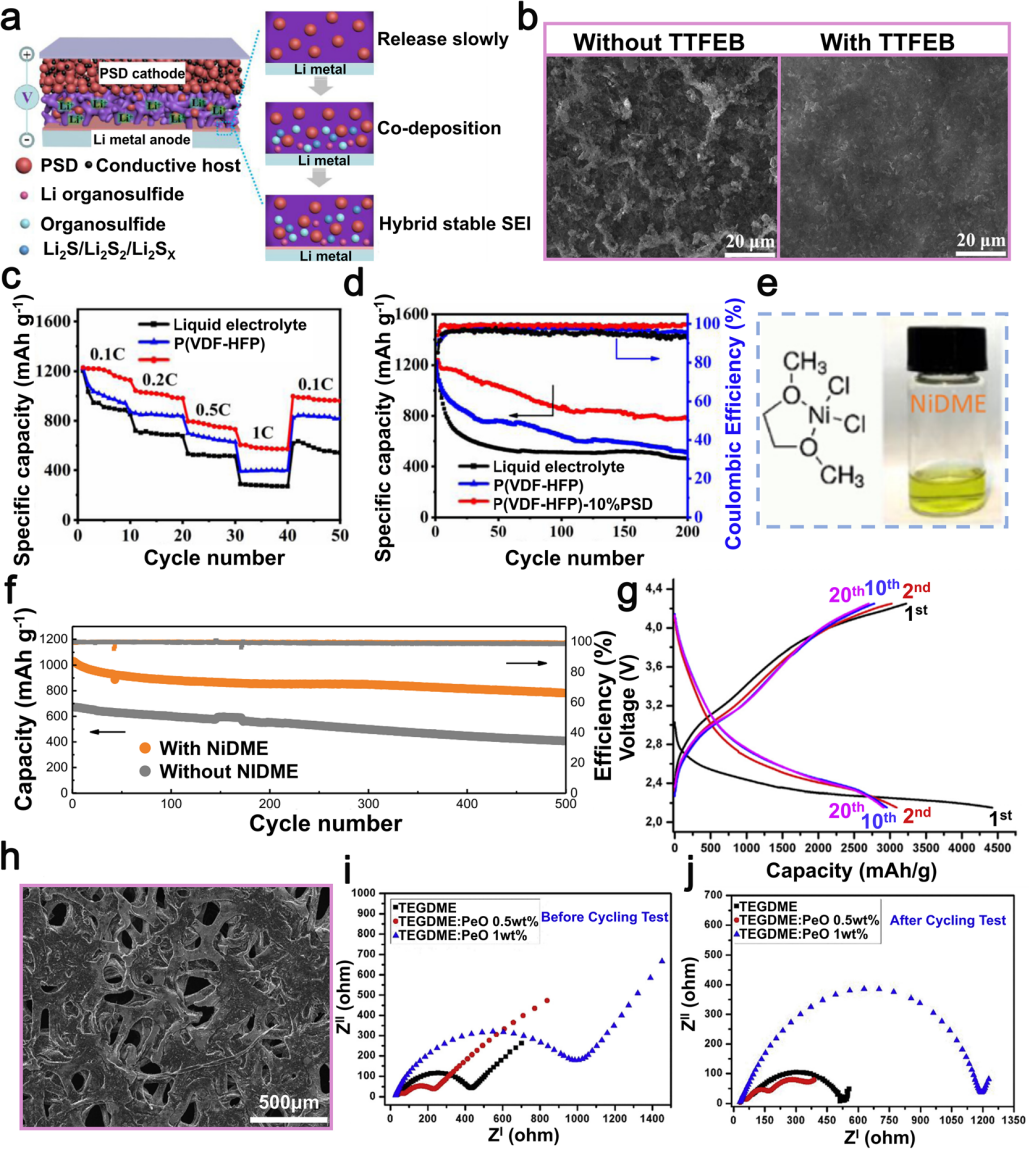
(a) Schematic of the interaction mechanism between P(VDF-HFP) and PSD and Li metal. (b) SEM images of the Li anode with/without 10% PSD additive after 200 cycles at 0.1C. (c) Rate performance of Li–S batteries under different electrolytes. (d) Cycling performance of Li–S batteries under different electrolytes. Reproduced with permission from ref. 164. Copyright 2019, Elsevier. (e) Chemical structure of NiDME. (f) Cycling stability of Li–S batteries with/without the NiDME additive. Reproduced with permission from ref. 165. Copyright 2020, Elsevier. (g) Capacity–voltage curves measured with 1.0 wt% PVDF in 1 M TEGDME/LiPF_6_ electrolyte. (h) SEM images of the cathode surface of Li–O_2_ batteries with 1.0 wt% PVDF. Electrochemical impedance spectra (EIS) (i) before and (j) after cycles with PVDF. Reproduced with permission from ref. 166. Copyright 2015, Elsevier.

In 2019, Jiang *et al.* utilized poly(sulfur-1,3-diisopropenylbenzene) (PSD) as both a cathode material and an additive within the poly(vinylidene fluoride-hexafluoropropylene) (P(VDF-HFP)) polymer electrolyte.^164^ The research findings indicate that the incorporation of 10% PSD into P(VDF-HFP) polymer electrolyte at room temperature leads to a peak ionic conductivity of 2.27 × 10^−3^ S cm^−1^. This polymer electrolyte, when combined with PSD, has the ability to create a long-lasting and reliable organic/inorganic hybrid SEI layer on lithium metal (Fig. 16a). By employing the Li–S battery featuring a 10% PSD, it can be observed that following 200 cycles at a rate of 0.1C, there is a noticeable improvement in the smoothness of the lithium metal anode surface, accompanied by a significant reduction in dendrite formation (Fig. 16b). The rate capability and cycle stability of the Li–S batteries with quasi-solid-state electrolyte, composed of PSD and P(VDF-HFP)-based polymer, were found to be excellent. These batteries achieved discharge capacities of 613 mAh g^−1^ at a rate of 0.1C and 780 mAh g^−1^ at a rate of 1C (Fig. 16c and d).^164^ This underscores the pivotal role of PSD as an additive in advancing high-performance Li–S batteries. In 2020, Chong Luo *et al.* successfully mitigated shuttle effect of sulfur by dissolving nickel chloride dimethoxyethane adduct (NiDME) (Fig. 16e) as an additive in the electrolyte.^165^ NiDME effectively captures LiPSs in the electrolyte, evenly accelerates redox reactions, and regulates the uniform and rapid deposition of Li_2_S. Surprisingly, the Li–S battery exhibited an impressive capacity retention of 784 mAh g^−1^ after undergoing 500 cycles at a rate of 1C, with a minimal decay of only 0.05% per cycle (Fig. 16f), achieved by incorporating merely 0.5 wt% of NiDME.^165^ This enhancement in performance is attributed to the additive accelerating the conversion of LiPSs, substantially lowering the activation energy (*E*_a_) of sulfur oxidation–reduction reactions, especially during the deposition of Li_2_S, and significantly reducing the accumulation of LiPS intermediates. In conclusion, the incorporation of NiDME as an electrolyte additive conclusively mitigates the electrolyte demands in practical Li–S batteries and significantly prolongs their life cycle.

In recent years, there has been significant research and focus on lithium–air batteries due to their potential energy density being similar to that of gasoline.^224,225^ However, the practical implementation of this technology has been consistently hindered by the continuous degradation of all components of the O_2_ electrode, such as solvents, lithium salts, binders, and carbon materials, throughout the cycling process.^226,227^ In 2015, A. Akbulut Uludag *et al.* successfully developed lithium–air batteries with graphene/30 wt% α-MnO_2_ air cathodes and lithium metal anodes by incorporating 1 wt% of polyvinylidene fluoride (PVDF) in TEGDME/LiPF_6_ electrolyte.^166^ Electrochemical experiments demonstrated that the incorporation of 1 wt% PVDF greatly improves the durability and cycling efficiency of the battery. Li–O_2_ batteries containing 1 wt% PVDF demonstrated impressive discharge capacities of 4488 mAh g^−1^ and 2992 mA h^−1^ after the first and twentieth cycles, respectively (Fig. 16g).^139^ The incorporation of PVDF effectively mitigated the accumulation of solid Li_2_O_2_ on the porous cathode (Fig. 16h), thereby preventing the obstruction of air passages by reaction products and facilitating oxygen diffusion at the electrode/air interface. After the addition of the PVDF polymer additive, the *R*_ct_ (charge transfer resistance) values were reduced, leading to intensified reactions between the electrolyte and the electrodes,^228,229^ with the lowest *R*_ct_ values observed for TEGDME/LiPF_6_ electrolytecontaining PVDF before cycling (Fig. 16i and j). Furthermore, this polymer additive, which possesses potent electron-withdrawing functional groups, facilitates the dissolution of lithium peroxide generated during the discharge process. As a result, it effectively safeguards the stability and integrity of the lithium anode.^230^ Thus, PVDF polymer as an additive not only demonstrates superior discharge performance but also exhibits remarkable stability.

In solid-state lithium metal batteries, limited dissociation efficiency of lithium salts and unstable (Li(DMF)_*x*_)^+^ solvated structures significantly hinder the ionic transport and stability of the electrolyte–electrode interface.^167^ To address this challenge, Zhao *et al.* introduced a sulfonylated chitosan (SCS)-modified poly(vinylidene fluoride) (PVDF-SCS) electrolyte in 2025.^231^ The nitrogen-based anion receptor in SCS preferentially disrupts cation–anion pairs, effectively increasing the concentration of free Li-ions. Additionally, the electron-deficient nitrogen sites within SCS strongly coordinate with lithium salt anions (*e.g.*, FSI^−^), facilitating their electrochemical reduction and the formation of a robust, inorganic-rich SEI. This unique anion-modulated mechanism boosts the ionic conductivity of Li-ions in the PVDF-SCS electrolyte up to 1.35 mS cm^−1^, effectively suppressing lithium dendrite growth and ensuring lithium metal anode stability. In practical Li|PVDF-SCS|NCM523 full batteries, the batteries exhibited stable cycling over 400 cycles, even under a high voltage of 4.3 V, demonstrating remarkable cycle stability. These results highlight that employing anion-engineering strategies to simultaneously enhance Li-ion transport and interfacial stability presents a promising approach for the development of high-performance solid-state lithium metal batteries.^232,233^

Polymer-based electrolyte additives have shown great promise in enhancing LMB performance by improving the structural and electrochemical stability of the SEI. High-molecular-weight polymers, such as PAN, OP-10, and HPMC, provide excellent mechanical robustness, thermal stability, and dendrite resistance. PAN's nitrile groups (–C≡N) facilitate Li-ion transport and form Li_3_N-rich SEI layers, significantly reducing interfacial impedance and suppressing dendrite growth. OP-10, containing EO interconnected by phenyl chains, promotes uniform lithium deposition and the formation of a flexible, durable SEI, resulting in minimal capacity fading and prolonged cycle life over 1000 cycles. GPEs modified with biodegradable polymers such as HPMC further enhance ionic conductivity and mechanical strength, demonstrating excellent cycling stability and improved lithium deposition uniformity. Incorporation of PST as an additive in Li–S batteries generates a robust hybrid SEI composed of organic polysulfides and inorganic Li_2_S/Li_2_S_2_, achieving outstanding coulombic efficiency (≈99%) and stable capacity retention across hundreds of cycles. Innovative zwitterionic electrolyte additives, such as 3-(triethylammonium)-propane-1-sulfonate (TEAPS), have been introduced to stabilize both cathode and anode interfaces simultaneously.^234,235^ TEAPS promotes the formation of a robust fluorine/sulfur/nitrogen-rich SEI and CEI, substantially improving cycling performance at high voltages. In solid-state systems, PVDF-SCS electrolyte leverages anion engineering to enhance lithium salt dissociation and form stable, inorganic-rich SEIs. This approach yields significantly elevated Li-ion conductivity (up to 1.35 mS cm^−1^) and robust interfacial stability, enabling stable operation for over 400 cycles even under high-voltage conditions (4.3 V). These findings collectively underscore the critical role of polymer-based additives in enhancing the electrochemical performance and interface stability of lithium metal batteries, highlighting their substantial potential for advancing next-generation energy storage technologies.

## 5. Conclusions and outlook

Compared to inorganic additives, organic additives possess higher solubility, enhanced adaptability, and abundant functional groups. These characteristics have rendered them highly advantageous when applied to LMBs and have sparked immense interest in their potential to improve battery performance as electrolyte additives. Up to now, the developmental research on organic additives as electrolyte additives has achieved considerable success. Below is a brief overview of the impact of organic additives, when used as electrolyte additives, on the electrochemical efficiency of LMBs, as depicted in Tables 1 and 2.

(1) Ester compounds as electrolyte additives. Ester additives are predominantly employed in carbonate-based electrolytes due to their strong polarity, excellent compatibility, and their ability to form stable and fluorine-rich SEI layers in polar solvent environments. Some ester compounds, due to their high dipole moments and hydrophobic functional groups, assist in forming a stable and hydrophobic SEI layer. This not only improves the battery's cycle stability but also protects the lithium metal anode. Some ester compounds also aid in forming lithium-friendly bonds and compounds, thereby inhibiting lithium dendrite formation and improving electrochemical performance. The fluorine-containing functional groups possessed by some ester compounds can help form an SEI layer rich in LiF and enhance the transport rate of Li-ions in the electrolyte, thereby achieving uniform Li deposition. The growth of lithium dendrites is effectively suppressed, thereby safeguarding the lithium metal anode and significantly enhancing the safety of LMBs.

(2) Organic sulfides as electrolyte additives. Organic sulfide additives often demonstrate superior effectiveness in ether-based electrolytes, especially in Li–S battery systems, due to their unique ability to modulate Li-ion solvation structures and interactions with polysulfides. Some organic sulfides have the ability to modify the solvation sheath structure of Li-ions, enabling a homogeneous deposition of Li-ions and facilitating the formation of a durable and secure SEI layer. Some small molecules containing sulfide compounds hinder the formation of Li dendrites by impeding the degradation of valuable constituents within the electrolyte, thereby augmenting the longevity of cycling. Some organic sulfides ensure the integrity of the lithium anode surface by accelerating the conversion of polysulfides and inhibiting the corrosion of the lithium anode.

(3) Carboxylic acid compounds as electrolyte additives. Carboxylic acid compound additives are predominantly applied in carbonate-based electrolytes due to their capability to facilitate robust SEI formation *via* controlled decomposition reactions at lithium metal interfaces. Carboxylic acid compounds are cost-effective and facilitate the formation of a lithium-containing SEI layer by facilitating the thorough decomposition of anions at the negative electrode. This results in a more uniform and dense deposition of lithium. At the same time, it also achieves reversible lithium metal plating–stripping, thus exhibiting high coulombic efficiency. Some carboxylic acid compounds can enhance the conductivity of the electrolyte by controlling the transport of Li-ions through the creation of a consistent and enduring SEI.

(4) Sulfonic acid compounds, alcohol compounds, and phenol compounds as electrolyte additives. Sulfonic acid, alcohol, and phenol compound additives typically exhibit exceptional performance in ether-based and carbonate-based electrolytes due to their low molecular weight, high polarity, and strong affinity toward lithium ions, enhancing their compatibility and SEI-forming properties. Given the distinctive molecular structure and low molecular weight of sulfonic acid compounds, it is common for them to exhibit remarkable solubility and efficient ion conduction capabilities within electrolytic solutions. This promotes a more consistent migration of Li-ions between the electrolyte and lithium metal interface, resulting in a deceleration of lithium dendrite formation. The abundant functional groups can form strong electronic interactions with Li-ions, leading to a more stable and uniform SEI layer, thereby improving battery stability. As compounds containing alcohol, small molecule compounds hinder the transportation of polysulfides (LiPSs) and form a consistent and enduring SEI layer *in situ* to inhibit the expansion of Li dendrites. The utilization of phenol compounds containing thiol functional groups enables the fabrication of organic SEI layers, which effectively shield the lithium anode against detrimental side reactions, minimize the consumption of pristine lithium and electrolyte, and prolong the battery's cycle life.

(5) Proteins as electrolyte additives. Protein additives are uniquely effective in aqueous and specialized solvent systems due to their natural functional groups and compatibility with diverse electrolyte chemistries, allowing targeted modification of the lithium anode surface. Proteins, as representatives of biological macromolecules, have many advantages that traditional polymer additives cannot match. Non-toxic proteins can reduce costs and environmental impact and enhance the safety of LMBs. Proteins have the ability to specifically and favorably bind to imperfections on the surface of a metal anode, causing changes in the distribution of electric fields in the surrounding area and preventing dendrite formation. The adsorption effect of proteins is enhanced by the distinctive alteration in their secondary structure when exposed to electrolytes. Furthermore, the inclusion of protein additives contributes to the development of the SEI by enhancing its mechanical characteristics and promoting superior ionic conductivity. These substances have the ability to specifically attach themselves to and mend fissures in the SEI while it undergoes cycling, thus ensuring sustained safeguarding for the metallic anode.

(6) Polymers as electrolyte additives. Polymer additives are frequently utilized in solid-state and GPEs because their macromolecular structures enable strong mechanical reinforcement of the SEI and stable interactions with lithium salts and lithium metal interfaces. Polymer macromolecules can form lithium compounds with lithium metal, significantly reducing interface impedance and enhancing battery stability. Specific functional groups in polymer additives can achieve uniform deposition of Li-ions through coordination with Li-ions. The formation of a strong and durable SEI layer by highly crosslinked polymers limits the exposure of electrolyte solvents to the anode surface, facilitating stable lithium stripping and plating over extended periods at high current densities. Some polymer additives have the ability to dissolve polysulfides and interact with Li metal, resulting in the creation of inorganic constituents that contribute to the development of SEI layers. This enhances the flexibility and stability of the SEI during plating/stripping operations, ultimately leading to an improvement in coulombic efficiency.

**Fig. 17:**
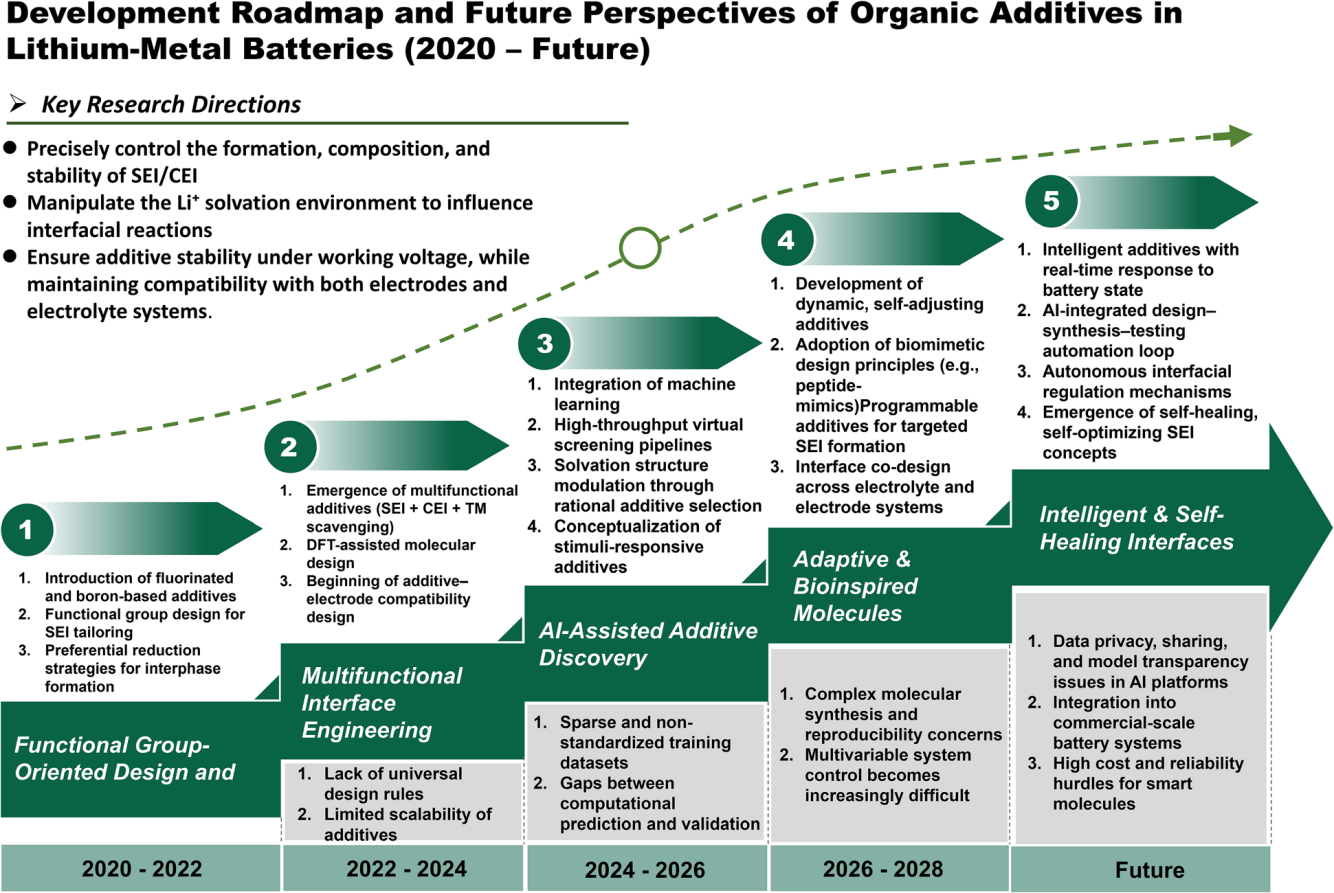
Future prospects and challenges of organic additives in LMBs.

Given the excellent properties of organic additives, it is evident that the application of organic substances as electrolyte additives in LMBs holds great promise for future research, development, and commercial application (Fig. 17a). Future advancements in organic electrolyte additives applied to LMBs can effectively focus on several key aspects:

(1) The unique molecular structures of organic additives, including functional group diversity, chain length, molecular weight, polarity, and steric configuration, significantly enhance their solubility, adaptability, and tailorability within various electrolyte systems. A multitude of functional groups such as ester, ether, sulfide, hydroxyl, amino, and carboxyl groups are particularly effective at modulating the formation, structure, and stability of SEI layer. A stable, uniform, and dense SEI layer directly contributes to more efficient lithium-ion transport, mitigates dendrite growth, and improves interfacial stability, thereby enhancing overall battery performance, energy density, and cycle life. Researchers should further explore structure–property relationships in organic additives, utilizing advanced computational modeling and *in situ* characterization techniques, to develop additives with optimized molecular architectures tailored specifically to enhance lithium metal battery performance.

(2) Although organic additives have demonstrated significant benefits in improving battery performance and protecting lithium metal anodes, their interaction and compatibility with cathode materials remain insufficiently understood. It is crucial to investigate how organic additives influence cathode–electrolyte interfaces, electrolyte oxidation stability, and interfacial impedance changes at cathode surfaces. Comprehensive studies focusing on additive compatibility across electrode pairs will allow the development of multifunctional additives capable of simultaneously enhancing the performance and stability of both anodes and cathodes. This holistic approach will drive the advancement of high-energy-density lithium batteries with balanced electrochemical performance.

(3) Transitioning the effectiveness of organic additives from laboratory research to industrial application presents multiple challenges. Ensuring scalability, maintaining consistent performance, and safeguarding battery safety under industrial manufacturing conditions require extensive evaluation and optimization. Future efforts should emphasize testing organic additives under realistic, large-scale production scenarios, assessing long-term cycling stability, rate performance, thermal stability, and safety under abuse conditions. Collaborative efforts between academia and industry will be essential in bridging the gap from laboratory demonstrations to commercial deployment, thereby facilitating the practical implementation and commercialization of advanced LMB technologies.

(4) Organic additives with simpler molecular structures typically offer advantages in terms of easier synthesis, lower production costs, and greater scalability for mass production. Additionally, the growing emphasis on sustainability and environmental stewardship underscores the urgent need to develop biodegradable, non-toxic, and environmentally friendly organic additives. Future research directions should prioritize the exploration and synthesis of bio-derived organic additives, recyclable additive systems, and green chemical processes. These environmentally sustainable additives will help mitigate the ecological footprint associated with battery manufacturing and disposal, aligning battery development with global environmental protection goals.

(5) Emerging technologies such as artificial intelligence (AI), machine learning (ML), and advanced materials informatics will play significant roles in accelerating the discovery and optimization of organic electrolyte additives. Integrating these computational tools with high-throughput experimentation and data-driven approaches can rapidly identify promising additive candidates, predict their performance, and systematically optimize additive formulations and electrolyte systems. Leveraging these advanced techniques will greatly streamline the innovation process, reducing development timelines, and enhancing the overall efficiency of research in organic electrolyte additives.

In the forthcoming years, organic additives will undoubtedly play a pivotal role in revolutionizing energy storage technologies. We firmly believe that continued exploration and refinement of organic electrolyte additives will significantly drive advancements in lithium metal battery performance, promoting sustainable growth and innovation in energy storage and renewable energy sectors.

## Author contributions

All authors contributed to the preparation of the manuscript. Wei Gu, lead author, contributed to the conceptualization, research, and writing of the manuscript. Di He and Yuting Qin collected references. Chongchong Fu and Jiahui Lu participated in the image design. Guoxiu Wang provided overall guidance and oversight of the research process. Tianyi Wang and Bing Sun, corresponding author, provided supervision, critical review, and final approval of the manuscript.

## Conflicts of interest

There are no conflicts to declare.

## Data Availability

No primary research results, software or code have been included and no new data were generated or analysed as part of this review.
